# Proteolytic Origin of the Soluble Human IL-6R In Vivo and a Decisive Role of N-Glycosylation

**DOI:** 10.1371/journal.pbio.2000080

**Published:** 2017-01-06

**Authors:** Steffen Riethmueller, Prasath Somasundaram, Johanna C. Ehlers, Chien-Wen Hung, Charlotte M. Flynn, Juliane Lokau, Maria Agthe, Stefan Düsterhöft, Yijue Zhu, Joachim Grötzinger, Inken Lorenzen, Tomas Koudelka, Kosuke Yamamoto, Ute Pickhinke, Rielana Wichert, Christoph Becker-Pauly, Marisa Rädisch, Alexander Albrecht, Markus Hessefort, Dominik Stahnke, Carlo Unverzagt, Stefan Rose-John, Andreas Tholey, Christoph Garbers

**Affiliations:** 1 Institute of Biochemistry, Kiel University, Kiel, Germany; 2 Systematic Proteomics & Bioanalytics, Institute for Experimental Medicine, Kiel University, Kiel, Germany; 3 Institute of Biochemistry, Unit for Degradomics of the Protease Web, Kiel University, Kiel, Germany; 4 Bioorganic Chemistry, Gebaeude NWI, University of Bayreuth, Bayreuth, Germany; National Jewish Health, United States of America

## Abstract

Signaling of the cytokine interleukin-6 (IL-6) via its soluble IL-6 receptor (sIL-6R) is responsible for the proinflammatory properties of IL-6 and constitutes an attractive therapeutic target, but how the sIL-6R is generated in vivo remains largely unclear. Here, we use liquid chromatography–mass spectrometry to identify an sIL-6R form in human serum that originates from proteolytic cleavage, map its cleavage site between Pro-355 and Val-356, and determine the occupancy of all O- and N-glycosylation sites of the human sIL-6R. The metalloprotease a disintegrin and metalloproteinase 17 (ADAM17) uses this cleavage site in vitro, and mutation of Val-356 is sufficient to completely abrogate IL-6R proteolysis. N- and O-glycosylation were dispensable for signaling of the IL-6R, but proteolysis was orchestrated by an N- and O-glycosylated sequon near the cleavage site and an N-glycan exosite in domain D1. Proteolysis of an IL-6R completely devoid of glycans is significantly impaired. Thus, glycosylation is an important regulator for sIL-6R generation.

## Author Summary

Interleukin-6 (IL-6) is a cytokine secreted by our body upon infection or trauma to stimulate the immune system response. IL-6 is partially responsible for fever and triggers inflammation in many diseases. It activates its target cells via the membrane-bound IL-6 receptor (IL-6R), and soluble forms of this receptor (sIL-6R) are present in high amounts in the serum of healthy individuals and mediate the inflammatory response in all cells of the human body. However, it remains unclear how the soluble form of this cytokine is generated in humans. In this study, we isolate sIL-6R from human serum and show that the majority is produced via cleavage of the membrane-bound IL-6R by a protease. We identify the exact cleavage site and find that it is identical to a cleavage site used by the metalloprotease ADAM17. We further show that glycosylation, a post-transcriptional modification, is dispensable for the transport and biological function of IL-6R and map the occupancy of all O- and N-glycosylation sites. However, we find that only a single N-glycan is critically involved in the regulation of proteolysis by ADAM17 and conclude that glycosylation is an important regulator for sIL-6R generation.

## Introduction

Interleukin-6 (IL-6) is a pleiotropic cytokine with important functions in many physiological and pathophysiological conditions [1, 2]. IL-6 activates intracellular signaling cascades through a homodimer of the ubiquitously expressed β-receptor glycoprotein 130 (gp130) [3] but first has to bind to its nonsignaling alpha receptor (IL-6R). The IL-6R is expressed in a cell- and tissue-specific manner and only found on hepatocytes and some leukocytes like neutrophils and T cells [3–5]. Signaling via the membrane-bound IL-6R is mostly regenerative and anti-inflammatory [5, 6].

The IL-6R is a typical type-I transmembrane protein that consists of an immunoglobulin (Ig)-like domain (“D1”), the cytokine-binding module (CBM) residing in two fibronectin-type-III domains (“D2” and “D3”), and a 55 amino-acid residue-long flexible stalk region that is followed by a transmembrane and an intracellular region. A minimal length of 22 amino acids of the stalk region is required for efficient IL-6 classic signaling, which corresponds to a stalk length of approximately 83 Å [7].

N-linked glycosylation is an important post-translational modification that can ensure correct folding and stability of a protein [8]. It has been shown that N-linked glycosylation of gp130 is essential for its stability but dispensable for the signaling function [9]. However, other signaling receptors require glycans for the ability to bind their respective ligands, e.g., the receptors for epidermal growth factor (EGF) [10], granulocyte-macrophage colony-stimulating factor (GM-CSF) [11], or C-X-C motif chemokine 12 (CXCL12) [12]. Although some asparagine residues within the extracellular part of the IL-6R have been identified that are used for N-linked glycosylation in vitro [13, 14], no functional role for these modifications has been determined. Addition of carbohydrates to serine or threonine residues, called O-linked glycosylation, has not been described so far for the IL-6R.

Soluble forms of the IL-6R (sIL-6R) are found in human serum at concentrations of about 30–70 ng/ml. IL-6 binds to membrane-bound and sIL-6R with similar affinity, and signaling of IL-6/sIL-6R has been termed IL-6 trans-signaling and is causative for the proinflammatory properties of IL-6. Specific inhibition of IL-6 trans-signaling holds the promise to be as effective as total IL-6 blockade, but with reduced side effects like enhanced susceptibility to bacterial infections [15]. One mechanism that contributes to sIL-6R generation is alternative splicing of the *IL6R* mRNA, which results in a unique C-terminus of ten amino-acid residues [16], and only this form of the sIL-6R has been detected in human serum [17]. However, only 1%–20% of the total sIL-6R is generated by alternative splicing [18–21], and the generation mechanism of the majority remains unknown. Recently, we described expression of the IL-6R on circulating microvesicles, but this also accounted only for a minor proportion of the total sIL-6R [22]. In vitro, sIL-6R can be efficiently generated by limited proteolysis, predominantly by the metalloproteases ADAM10 and ADAM17.

ADAM17-mediated shedding can be induced by a variety of stimuli, and the phorbol ester phorbol-12-myristate-13-acetate (PMA) is the strongest known activator in vitro. Carboxypeptidase treatment of sIL-6R, which was purified from the supernatant of PMA-treated IL-6R overexpressing COS-7 cells, led to the identification of the ADAM17 cleavage site within the IL-6R between Gln-357 and Asp-358 [14]. However, cleavage site profiling revealed that glutamine and aspartic acid are rather disfavored at the P1 and P1′ positions [23], and the IL-6R is the only ADAM17 substrate with such a cleavage site that is listed in the MEROPS database [24]. Indeed, cleavage of an IL-6R peptide with recombinant ADAM17 occurred between Pro-355 and Val-356 [25]. Nevertheless, deletion of amino-acid residues Ser-353 to Val-362 within the IL-6R stalk prevented ADAM17-mediated shedding and did not compromise its biological activity [7, 14]. Proteolysis by ADAM10, the closest homologue of ADAM17, still occurred, which suggested the usage of multiple cleavage sites by ADAM10 or different cleavage sites by the two proteases [7]. Besides the cleavage site, which is considered to be the major determinant of substrate/protease specificity, exosites are also known to contribute to the regulation of IL-6R proteolysis [26–28].

In this study, we identify by liquid chromatography–mass spectrometry (LC-MS) an sIL-6R form in human serum that is generated via limited proteolysis, map its cleavage site, and characterize the occupancy of all O- and N-glycosylation sites. We confirm that the same cleavage site is used by ADAM10 and ADAM17 in vitro and show that mutation of Val-356 at P1′ is sufficient to completely block proteolysis. We further show that glycosylation is dispensable for IL-6R trafficking and signaling but regulates proteolysis through an N-glycan exosite in domain D1 and a sequon that is both N- and O-glycosylated adjacent to the cleavage site.

## Results

### The Majority of sIL-6R in Human Serum Is Not Generated by Alternative mRNA Splicing

**Fig 1:**
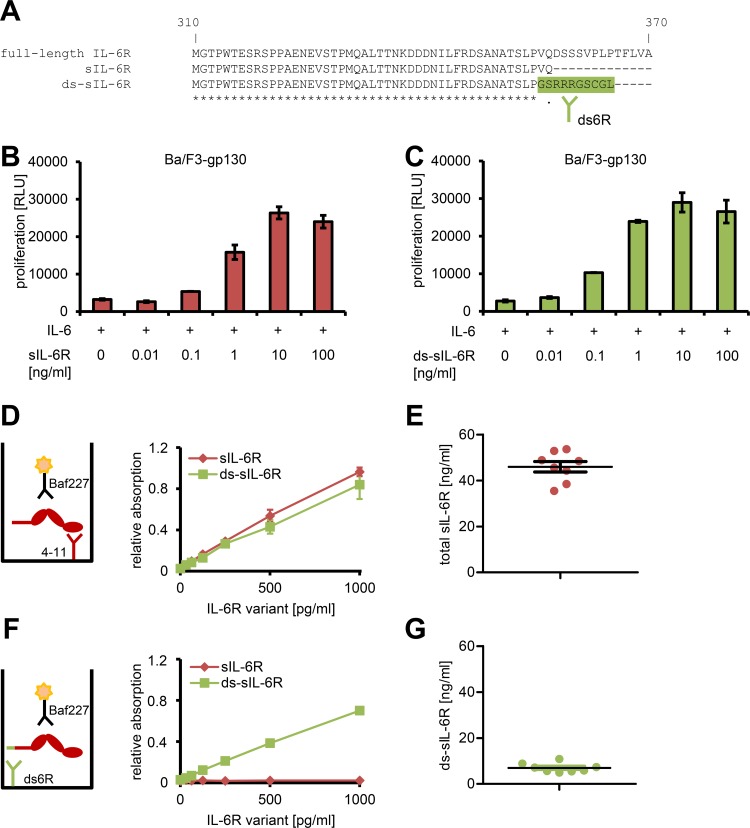
The majority of sIL-6R in human serum is not generated by alternative mRNA splicing. (A) Multiple sequence alignment of amino acids Met-310 to Ala-370 of the full-length human IL-6R with the sIL-6R (Met-310 to Gln-357) and amino acids Met-310 to Leu-365 of the differentially spliced sIL-6R (ds-sIL-6R). The ten unique C-terminal amino-acid residues of the sIL-6R, which represent the epitope against which the antibody ds6R was raised, are colored in green. (B, C) Proliferation assay of Ba/F3-gp130 cells in response to 10 ng/ml IL-6 and increasing amounts (0–100 ng/ml) of either (B) recombinant sIL-6R or (C) recombinant ds-sIL-6R. Cell viability was assessed after 48 h. Data shown are the mean ± the standard deviation (SD) of one representative experiment with three biological replicates. (D) Schematic representation of the sandwich ELISA that recognizes all forms of the sIL-6R (left) and proof-of-principle experiment with recombinant proteins (mean ± SD, *n* = 3). (E) Determination of the total sIL-6R levels in the serum of eight healthy donors. Values for the mean ± the standard error of the mean (SEM) are indicated. (F) Schematic representation of the sandwich ELISA that recognizes only the ds-sIL-6R (left) and proof-of-principle experiment with recombinant proteins (mean ± SD, *n* = 3). (G) Determination of the ds-sIL-6R levels in the serum of the same eight healthy donors as in (E). The values for the mean ± SEM are indicated.

Although the existence of sIL-6R in human body fluids has been known for more than 25 y [29, 30], the mechanisms of its generation are largely unexplored. Müller-Newen et al. were able to purify an sIL-6R from human plasma that was generated by alternative splicing of the *IL6R* mRNA (termed ds-sIL-6R hereafter) [17]. However, several studies using ELISA revealed that only 1%–20% of the total sIL-6R is generated by splicing [18–21], which suggests that other mechanisms must exist in parallel. We sought to confirm these findings and generated a polyclonal antibody (termed ds6R) against the ten unique C-terminal amino-acid residues of the ds-sIL-6R, which are not present in the full-length IL-6R or a proteolytically cleaved sIL-6R (Fig 1A). In order to be able to quantify total sIL-6R and to distinguish only ds-sIL-6R in human serum, we first expressed and purified both sIL-6R and ds-sIL-6R as recombinant proteins. As shown in Fig 1B and 1C, both sIL-6R forms were biologically active and equally well able to perform IL-6 trans-signaling, because they stimulated proliferation of Ba/F3-gp130 cells in a dose-dependent manner when combined with IL-6. A sandwich ELISA with a capture antibody that binds to the sIL-6R N-terminus (4–11) and a polyclonal detection antibody (Baf227, [21, 31]) measured recombinant sIL-6R and ds-sIL-6R with equal efficiency (Fig 1D) and allowed us to measure total sIL-6R levels in the serum of eight healthy donors, which were in a similar range as described before (46.0 ± 6.4 ng/ml, Fig 1E, [21]). Using the ds-sIL-6R-specific antibody ds6R, we set up an ELISA that only detects ds-sIL-6R and not other sIL-6R forms (Fig 1F, [16]). When we analyzed the same eight serum samples for ds-sIL-6R, we detected only 7.0 ± 2.0 ng/ml, which accounts for 15.1% ± 2.8% of the total sIL-6R (Fig 1G). Thus, our results confirm previous studies that found that a fraction of the sIL-6R in humans is generated via alternative splicing but that the majority must originate from a different mechanism.

### Mass Spectrometry Identifies a sIL-6R Form with a Protease-Derived C-Terminus and a Novel O-Glycosylation Site

**Fig 2:**
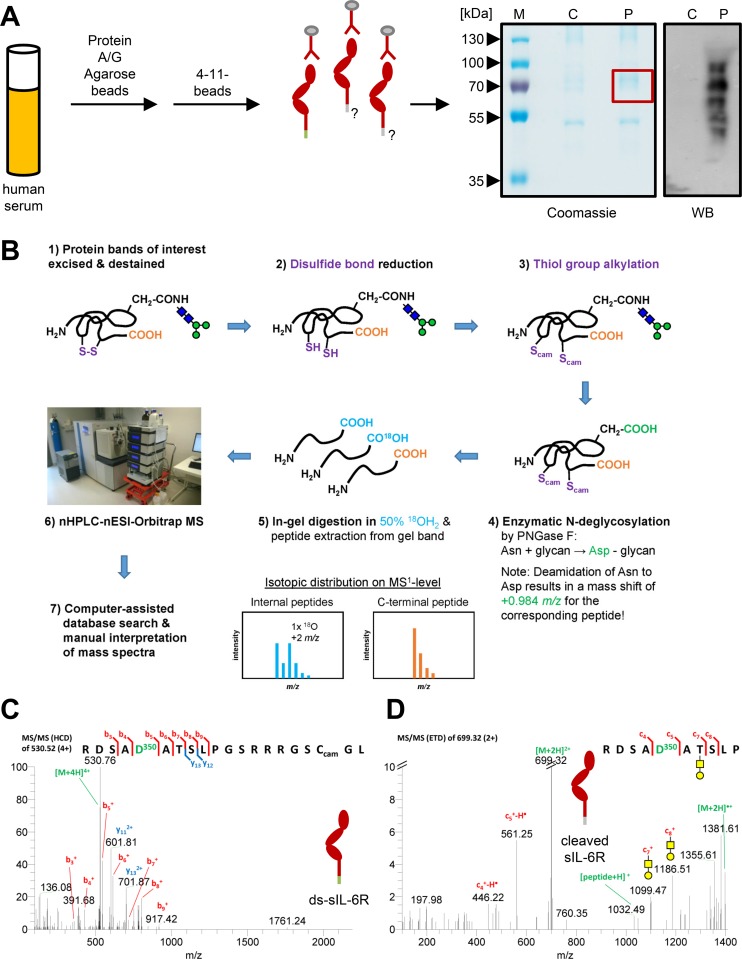
Identification of the ds-sIL-6R and a novel protease-derived sIL-6R from human serum via mass spectrometry (MS). (A) Schematic illustration of the procedure to precipitate total sIL-6R from human serum. A representative coomassie-stained sodium dodecyl sulfate (SDS) gel and a western blot of the precipitated protein are shown on the right. The SDS gels were run under nonreducing conditions (see Materials and Methods for details), and the region of the SDS gel excised for MS is indicated with a red box. The precipitated sIL-6R is detected with an antibody that specifically recognizes the ectodomain of the human IL-6R (4–11). (B) Proteomics workflow including disulfide bond reduction, thiol alkylation, enzymatic N-deglycosylation, proteolysis in presence of 50% H_2_^18^O, LC-MS/MS analysis, and data interpretation. (C) MS/MS spectrum (higher-energy collisional dissociation [HCD]) of the C-terminal peptide of the ds-sIL-6R identified via database searching. The N-glycan site Asn-350, which is modified to an Asp-350 because of PNGase F treatment, is shown in green. (D) MS/MS spectrum (electron-transfer dissociation [ETD]) of the C-terminal peptide of the protease-derived sIL-6R identified via database searching. The N-glycan site Asn-350, which is modified to an Asp-350 because of PNGase F treatment, is shown in green. One of the identified O-glycan structures on Thr-352 is shown.

In order to identify other human sIL-6R forms that exist in vivo, we performed an isotopic labeling strategy that is based on proteolysis in the presence of H_2_^18^O [32]. We isolated human serum, depleted the endogenous antibodies, and precipitated all sIL-6R forms by the sepharose-coupled 4–11 antibody, which binds to the N-terminal D1 domain (Fig 2A). We separated the precipitated proteins via SDS-PAGE under nonreducing conditions, confirmed sIL-6R presence in the precipitate via western blot, and excised the corresponding region of the coomassie-stained gel (Fig 2A). Using an anti-IL-6R antibody, we detected several proteins of different molecular weights, which most likely are different sIL-6R isoforms and complexes of these with other serum proteins that are not dissociated under nonreducing conditions (Fig 2A). Prior to in-gel proteolysis, the proteins were deglycosylated using peptide:N-glycosidase F (PNGase F). This endoglycosidase hydrolyzes the bond formed between the carbohydrate and the Asn side chain, resulting in the formation of an Asp residue at the former glycosylation site. Subsequently, protein digestion was performed in 50% H_2_^18^O-containing buffers. By using this method, all proteolytically generated *neo*-C-termini contain an ^18^O-isotope incorporated in the carboxyl group, while the original (canonical or truncated) protein C-terminus remains unmodified (Fig 2B).

We identified a peptide that maps to the unique C-terminus of the ds-sIL-6R (Fig 2C), which further corroborates that ds-sIL-6R contributes to the total amount of sIL-6R in human serum. Furthermore, we identified another C-terminal peptide, which ends with Pro-355 and thus could belong to an sIL-6R that is generated through proteolysis of the membrane-bound IL-6R between Pro-355 and Val-356 (Fig 2D). Both ds-sIL-6R and sIL-6R had an N-linked glycosylation at Asn-350, which was converted to Asp-350 during the N-deglycosylation with PNGase F (Fig 2C and 2D, colored in green). Furthermore, using electron transfer dissociation (ETD) MS/MS, we identified an O-glycosylation site within the Asn-350 sequon on Thr-352, which was decorated with several different glycan structures (Fig 2D and S1A–S1E Fig). With this MS-based strategy, we were for the first time able to identify an sIL-6R variant in human serum that does not originate from alternative splicing but appears to be generated by proteolysis.

### ADAM17 and ADAM10 Cleave the IL-6R between Pro-355 and Val-356 In Vitro

**Fig 3:**
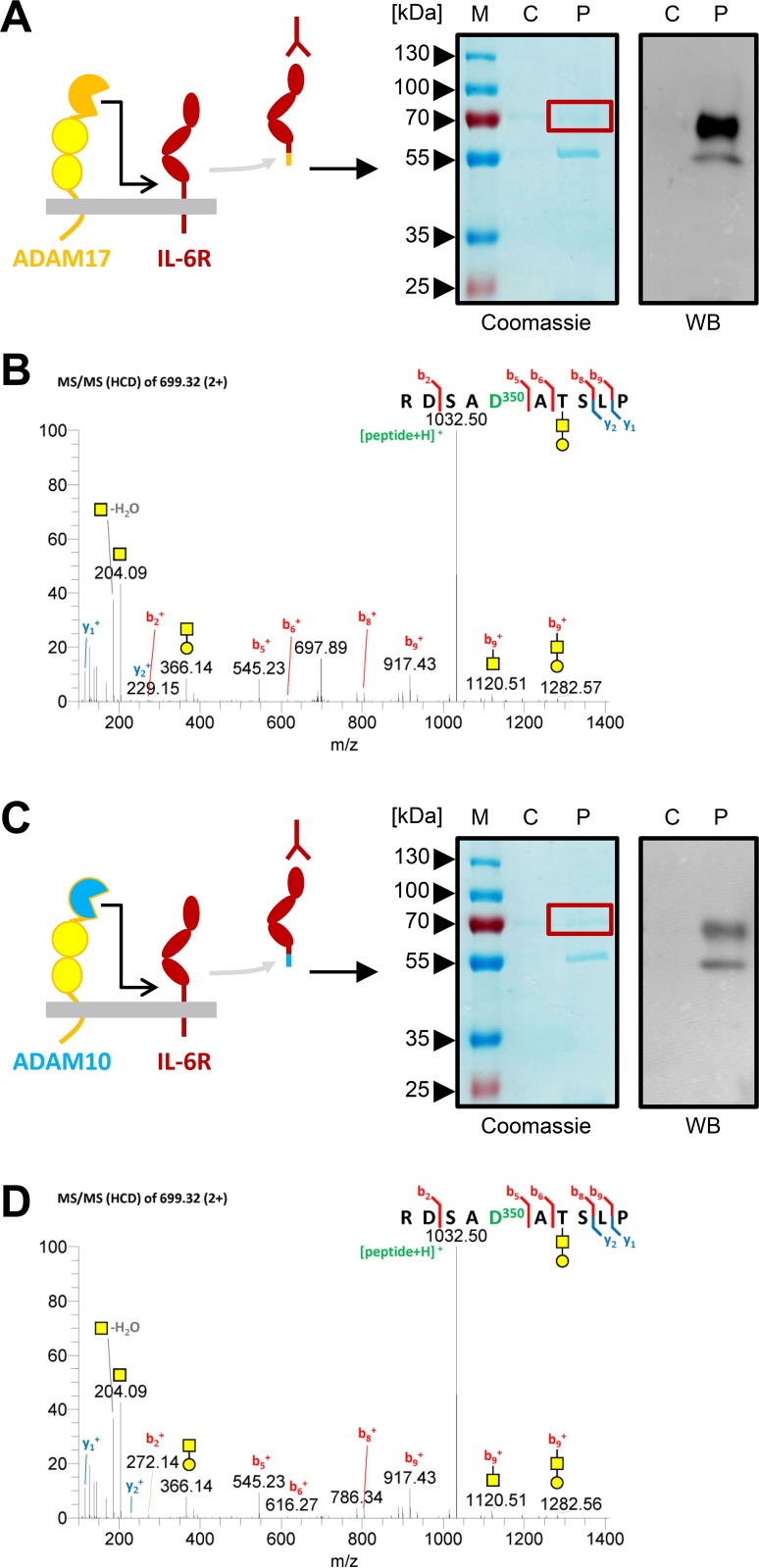
ADAM17 and ADAM10 cleave the IL-6R between Pro-355 and Val-356 in vitro. (A) A schematic illustration, a representative coomassie-stained SDS gel, and a western blot of the precipitated protein after PMA-induced ADAM17-mediated sIL-6R generation are shown. The SDS gels were run under reducing conditions (see Materials and Methods for details), and the region of the SDS gel excised for MS is indicated with a red box. The precipitated sIL-6R is detected with an antibody that specifically recognizes the ectodomain of the human IL-6R (4–11). (B) MS/MS spectra (HCD) of the C-terminal peptide of the sIL-6R identified by manual spectra interpretation. One of the identified O-glycan structures on Thr-352 is shown. (C) A schematic illustration, a representative coomassie-stained SDS gel, and a western blot of the precipitated protein after ionomycin-induced ADAM10-mediated sIL-6R generation are shown. The SDS gels and the western blot were performed as described under panel (A). (D) MS/MS spectra (HCD) of the C-terminal peptide of the sIL-6R identified by manual spectra interpretation. One of the identified O-glycan structures on Thr-352 is shown.

Having shown that a protease-derived sIL-6R exists in vivo, we sought to identify the protease that could be responsible for its generation. The IL-6R is a known substrate for three human proteases, the two metalloproteases ADAM10 and ADAM17 [7, 27, 33], and the neutrophil-derived serine protease cathepsin G (CG) [34, 35]. So far, the only protease of the IL-6R whose cleavage site has been mapped is ADAM17, which has been reported to cleave between Gln-357/Asp-358, two amino-acid residues downstream of Pro-355/Val-356 [14]. In order to recapitulate this finding, we transiently transfected HEK293 cells with a cDNA encoding human IL-6R and induced ADAM17-mediated shedding with PMA, a strong and well-known activator of ADAM17. We precipitated sIL-6R from the cell culture supernatant and performed SDS-PAGE and western blot (Fig 3A). The strongest band detected in the western blot corresponded to a cleavage product of the IL-6R, whereas the lower band at around 55 kDa most likely was the heavy chain of the antibody used for precipitation (Fig 3A). Indeed, MS analysis revealed a C-terminal peptide that ended with Pro-355 (Fig 3B), indicating exactly the same cleavage site that was used in vivo (Fig 2D). We could further confirm the N-glycan site on Asn-350 and the O-glycan site at Thr-352 within the same sequon, which was again decorated with the same pattern of glycan structures found in vivo (Fig 3B and S2A–S2E Fig). Thus, we could not verify the originally published cleavage site Gln-357/Asp-358 [14] but instead found an ADAM17 cleavage site between Pro-355/Val-356 that matches the published cleavage preferences of ADAM17 [23] and is in good agreement with other cleavage sites of ADAM17 substrates in the MEROPS database [24].

To identify the cleavage site for ADAM10, we performed a similar experiment and stimulated transiently transfected HEK293 cells with ionomycin, a calcium ionophore that induces IL-6R cleavage by ADAM10 (Fig 3C) [7, 21, 27, 36]. Similar to ADAM17-mediated shedding (Fig 3A), we detected cleaved IL-6R and a band of lower molecular weight, which most likely corresponded to the heavy chain of the antibody used for precipitation (Fig 3C). Surprisingly, we identified the same cleavage site between Pro-355 and Val-356 (Fig 3D) and the same pattern of O-glycans at Thr-352 (S3A–S3D Fig), indicating that both proteases share the same cleavage site.

We did not determine the cleavage site used by CG, as the resulting sIL-6R was significantly smaller compared to the sIL-6R cleaved by ADAM17 (S3E Fig), indicating that CG cleaved further upstream within the IL-6R stalk and thus cannot be the protease responsible for the generation of the steady-state sIL-6R serum levels.

### Mutation of Val-356 Is Sufficient to Block Proteolysis of the IL-6R and Its Asp358Ala Variant

**Fig 4:**
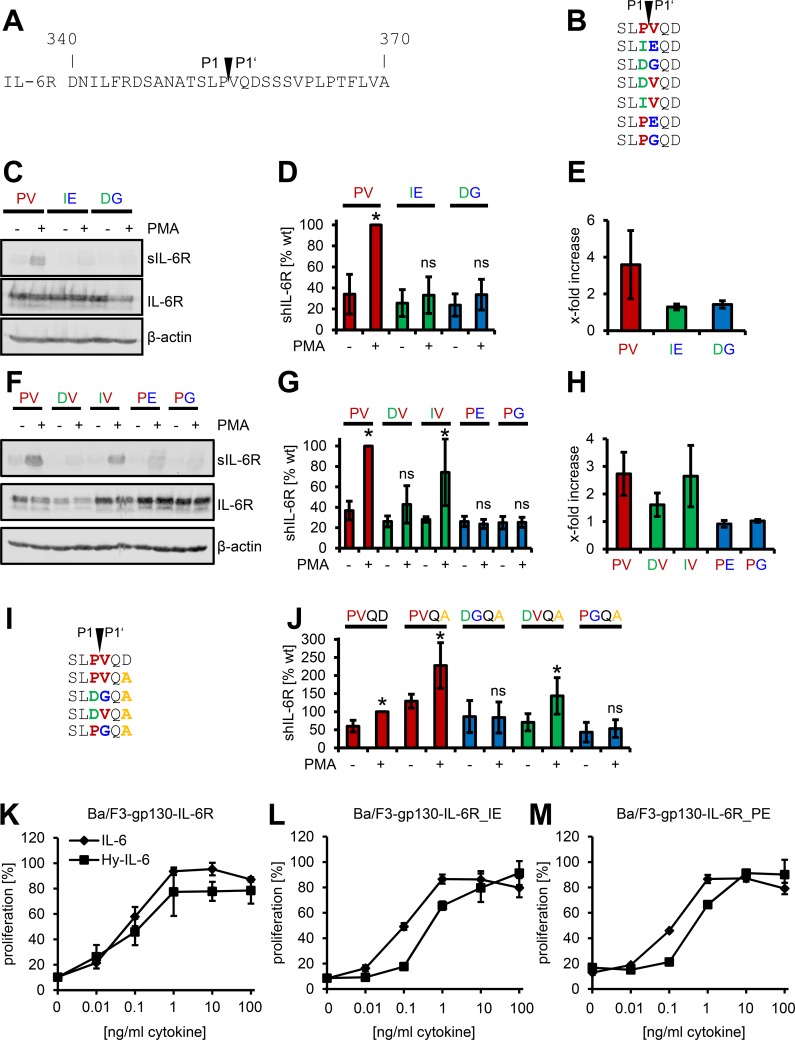
Mutation of Val-356 is sufficient to block proteolysis of the IL-6R and the Asp358Ala variant. (A) Amino-acid residues from Asp-340 to Ala-370 of the human IL-6R. The identified cleavage site between Pro-355 (P1) and Val-356 (P1′) is indicated. (B) Overview of the six different IL-6R cleavage site mutants. Mutations are colored in either green or blue; the amino-acid residues of the wild-type cleavage site are shown in red. (C) HEK293 cells were transiently transfected with expression plasmids encoding the wild-type IL-6R (PV) or the double mutants (IE, DG) as indicated. Cells were either treated with 100 nM PMA for 2 h or DMSO as vehicle control. sIL-6R was precipitated from the supernatant with concanavalin A-covered sepharose beads, and the cells were lysed. Both were analyzed via western blot, and β-actin served as the loading control. One out of three experiments with similar outcomes is shown. (D, E) The experiment was performed as described under (C), but sIL-6R generation was analyzed via ELISA. In (D), the amount of sIL-6R generated after PMA stimulation of the wild-type IL-6R (PV) was set to 100%, and all other values were calculated accordingly. In (E), the amount of sIL-6R without stimulation was considered as constitutive shedding and set to 1, and the increase of sIL-6R was calculated. Data shown are the mean ± SD from at least three independent experiments (*p < 0.05, ns = no significant difference). (F–H) HEK293 cells were transiently transfected with expression plasmids encoding the wild-type IL-6R (PV) or the single mutants (DV, IV, PE, PG) as indicated. The experiments were performed as described in (C) to (E). (I) Overview of the four different IL-6R cleavage site mutants of the IL-6R Asp358Ala variant. Mutations are colored in either green or blue, the amino-acid residues of the wild-type cleavage site are shown in red, and the Asp358Ala single nucleotide polymorphism (SNP) is colored in orange. (J) ADAM17-mediated proteolysis of the IL-6R variants depicted in (I) were analyzed as described in (D). (K–M) Equal numbers of Ba/F3-gp130 cells were incubated for 48 h with increasing amounts (0–100 ng/ml) of either IL-6 or Hyper-IL-6. The stably transduced IL-6R variant is indicated above the diagram. One representative experiment out of three performed is shown (mean ± SD, biological triplicates).

To further characterize the identified cleavage site, we created IL-6R variants with different point mutations at the P1 and the P1′ sites (Fig 4A and 4B). In general, we exchanged Pro-355 and Val-356 into amino-acid residues that have been shown to be disfavored by ADAM17 [23]. When we mutated the cleavage site to Ile-355/Glu-356 (termed IL-6R_IE hereafter; all other mutants are termed accordingly), we could not detect inducible formation of sIL-6R by western blot (Fig 4C) or ELISA (Fig 4D) after stimulation with PMA. The same was true for the IL-6R_DG mutant. When we calculated the increase in sIL-6R generation, the wild-type IL-6R was shed 3.6 ± 1.9-fold compared to vehicle-treated cells, whereas both mutants showed no increase in sIL-6R formation (1.3 ± 0.2-fold and 1.4 ± 0.2-fold, respectively, Fig 4E). We obtained similar results when we activated ADAM10 with ionomycin (S4A–S4C Fig). In order to determine if mutation of either P1 or P1′ alone would be sufficient to block ADAM-mediated proteolysis, we created the corresponding four single mutants (Fig 4B). Whereas IL-6R_DV and IL-6R_IV were still shed after PMA treatment, albeit to a minor extent compared to the wildtype (Fig 4F and 4G), mutation of the P1′ site (IL6R_PE and IL-6R_PG) completely abrogated proteolysis by ADAM17 (Fig 4F and 4G), and we could not detect any increase in sIL-6R formation (0.9 ± 0.1-fold and 1.0 ± 0.1-fold, Fig 4H). Similar results were again obtained for ADAM10-mediated cleavage (S4D–S4F Fig). In conclusion, our results show that mutation of the P1′ site (Val-356) is sufficient to completely block ADAM-mediated cleavage of the IL-6R.

The coding single nucleotide polymorphism (SNP) Asp358Ala within the IL-6R is located in close proximity to the cleavage site, and homozygous carriers of this SNP have increased sIL-6R serum levels [37, 38]. We have shown previously that the Asp358Ala (termed IL-6R_PVQA in this study) mutation renders the IL-6R more susceptible to ADAM-mediated cleavage [21]. In order to investigate whether mutation of the cleavage site would be sufficient to block this increased proteolysis, we created three additional IL-6R mutants that contained Asp358Ala combined with single or double mutations of the cleavage site (termed IL-6R_DGQA, IL-6R_DVQA, and IL-6R_PGQA; Fig 4I). First, we verified our previous finding [21] that IL-6R_PVQA was indeed shed more than the wild-type IL-6R_PVQD (Fig 4J). Mutation of both P1 and P1′ in IL-6R_DGQA completely abrogated PMA-inducible shedding, and mutation of P1′ alone, but not P1 alone, was sufficient to render the Asp358Ala mutant unresponsive towards proteolysis by ADAM17 (Fig 4J). Proteolysis by ADAM10 was also significantly reduced, although some inducible shedding by ionomycin could still be detected (S4G Fig). Thus, mutation of Val-356 is also sufficient to prevent the enhanced shedding of the Asp358Ala mutant.

Finally, we analyzed whether these mutations influenced the biological activity of the IL-6R. To this end, we stably transduced Ba/F3-gp130 cells with wild-type IL-6R and IL-6R mutants. After transduction, Ba/F3-gp130-hIL-6R cells proliferated in a dose-dependent manner (0–100 ng/ml) in response to IL-6 (Fig 4K). As an internal control, we incubated all cell lines with Hyper-IL-6, which is a fusion protein of IL-6 and the sIL-6R and thus activates proliferation via gp130 homodimerization independent of membrane-bound IL-6R. Ba/F3-gp130-IL-6R_IE, Ba/F3-gp130-IL-6R_PE, and Ba/F3-gp130-IL-6R_DG proliferated equally well compared to the wild-type cells, indicating that mutation of the cleavage site did not affect the biological function of the IL-6R (Fig 4L and 4M and S4H Fig).

### Determination of Four Additional N-Glycosylation Sites on the sIL-6R In Vitro and In Vivo via MS

**Fig 5:**
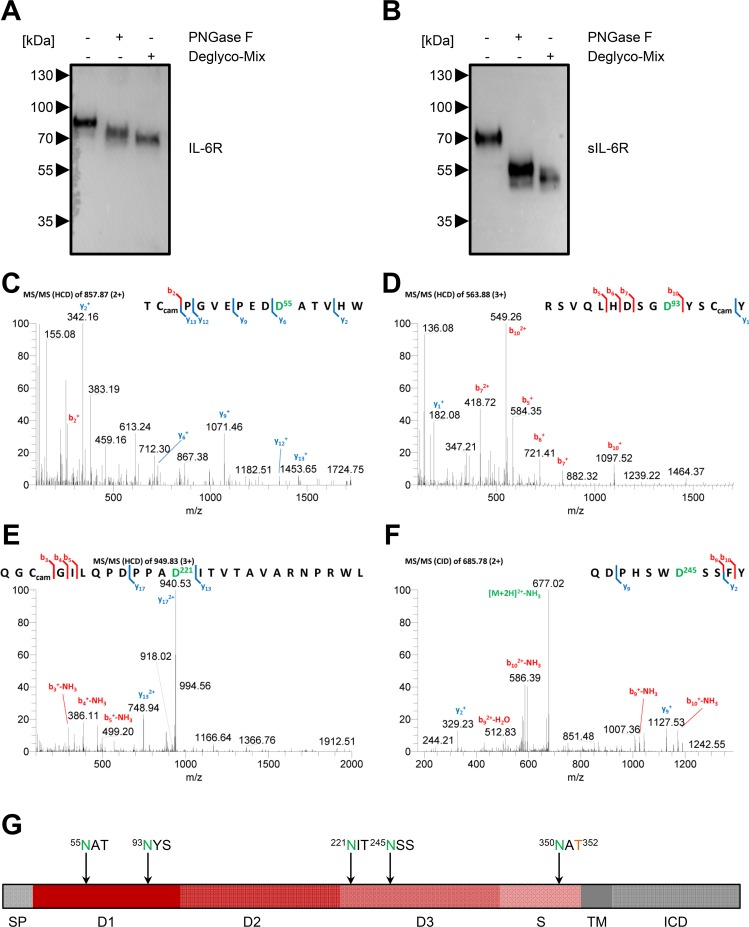
Determination of the N-glycosylation sites on the sIL-6R in vitro and in vivo via MS. (A) HEK293 cells were transiently transfected with an expression plasmid encoding IL-6R, and cells were lysed 48 h later. Lysates were treated with either PNGase F or Deglyco-Mix or were left untreated. Proteins were resolved on a 10% SDS gel and analyzed by western blot. One representative experiment out of two performed is shown. (B) HEK293 cells were transiently transfected with an expression plasmid encoding IL-6R, and cells were stimulated after 48 h with 100 nM PMA for 2 h. The sIL-6R was precipitated from the cell supernatant and afterwards treated and analyzed as described under panel (A). (C–F) MS/MS spectra (HCD) of the different peptides of the sIL-6R that contained N-linked glycans. The four glycosylation sites (Asn-55, Asn-93, Asn-221, and Asn-245) were modified to aspartic acid residues due to PNGase F treatment in the presence of H_2_^18^O-containing buffer and are shown in green. The use of Asn-350 as an N-glycosylation site was already shown in Fig 2C and 2D. Peptides were identified via database searching. (G) Schematic overview of the different domains of the IL-6R and the localization of the five N-glycosylation sites (green) and one O-glycosylation site (orange). D, domain; ICD, intracellular domain; S, stalk region; SP, signal peptide; TM, transmembrane region.

Although N-linked glycosylation of the IL-6R has been reported [13, 14], no functional role has been addressed so far. We first confirmed the presence of glycans on the full-length IL-6R and the sIL-6R by either removing just the N-linked glycans with PNGase F or by additionally also removing O-linked glycans (Fig 5A and 5B). While determining the cleavage site, we had already successfully detected an N-glycosylation site at Asn-350 and an O-glycosylation site at Thr-352, both in vitro and in vivo (Figs 2D and 3B). By performing N-deglycosylation in the presence of H_2_^18^O-containing buffers, we identified further N-glycosylation sites at Asn-55, Asn-93, Asn-221, and Asn-245 on sIL-6R derived from PMA-stimulated HEK293 cells (Fig 5C–5F). With the exception of Asn-245, we verified all N-glycosylation sites also on sIL-6R isolated from human serum (S5A–S5C Fig). As we could not detect any other O-glycosylation site in addition to Thr-352, the IL-6R contains two N-glycans located in domain D1, two N-glycans in domain D3, and a combined N-/O-glycan site within the stalk region adjacent to the cleavage site (Fig 5G).

### N- and O-Linked Glycosylation Are Dispensable for Transport, Signaling, and Cell-Surface Turnover of the IL-6R

**Fig 6:**
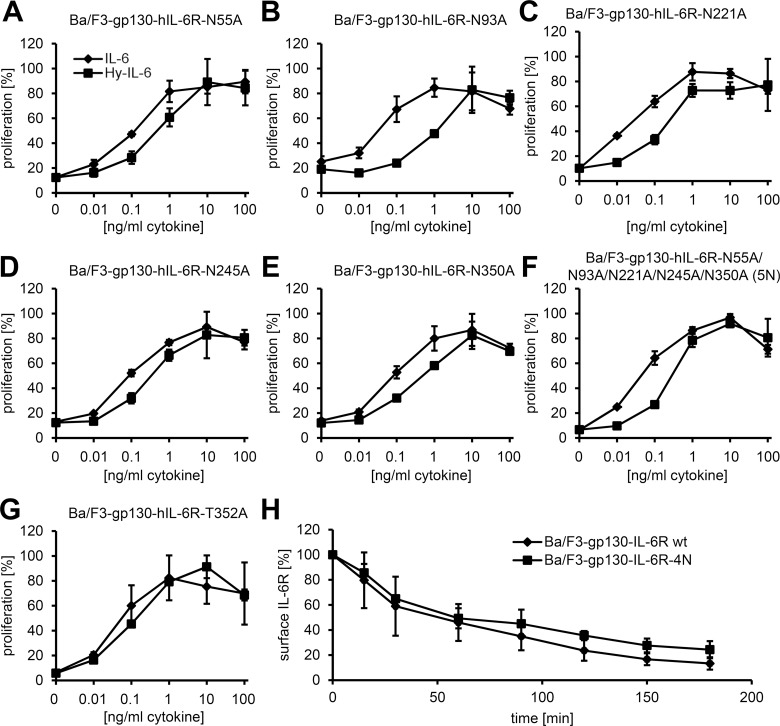
N- and O-linked glycosylation are dispensable for intracellular transport and signaling of the IL-6R. (A–G) Equal numbers of Ba/F3-gp130 cells were incubated for 48 h with increasing amounts (0–100 ng/ml) of either IL-6 or Hyper-IL-6. The stably transduced IL-6R variant is indicated above the diagram. One representative experiment out of three performed is shown (mean ± SD, biological triplicates). (H) Equal numbers of Ba/F3-gp130-hIL-6R and Ba/F3-gp130-hIL-6R-4N cells were labeled with an anti-IL-6R antibody and incubated at 37°C for the indicated time periods. The remaining cell-surface expression of the IL-6R was analyzed via flow cytometry. Data shown are the mean ± SD from three independent experiments.

In order to analyze a functional role for the determined glycans, we created IL-6R mutants that lacked either one, multiple, or all N-glycosylation sites and stably transduced Ba/F3-gp130 cells with these. Flow cytometry analysis revealed that all IL-6R mutants were transported to and expressed at the cell surface, albeit the amount slightly decreased when more N-glycans were deleted (S6A Fig). When we analyzed IL-6-dependent proliferation of the individual cell lines, we could not detect a difference between the wild-type IL-6R (Fig 4K), IL-6R mutants lacking one of the five N-glycosylation sites (Fig 6A–6E), IL-6R mutants lacking two (termed 2N), three (3N), or four N-glycosylation sites (4N, S6B–S6D Fig), or the IL-6R mutant that lacked all five N-glycans (5N, Fig 6F). Similarly, mutation of the O-glycosylation site on Thr-352 had no influence on IL-6 signaling (Fig 6G).

Finally, we analyzed protein stability and turnover at the cell surface of the wild-type IL-6R compared to the 4N mutant. For this purpose, we used a pulse-chase approach and labeled the IL-6R on the cells in the cold, shifted the cells back to 37°C for different time periods, and analyzed the remaining IL-6R on the cell surface via flow cytometry. As shown in Fig 6H, the wild-type IL-6R disappeared from the cell surface in a time-dependent manner (t_1/2_ = 44.1 ± 8.1 min), which did not differ significantly from the 4N mutant (t_1/2_ = 40.9 ± 5.2 min). Collectively, our data show that glycosylation of the IL-6R is not needed for transport to and expression at the cell surface, is dispensable for IL-6-mediated signal transduction, and does not alter the half-life of the IL-6R at the cell surface.

### The N-/O-Glycosylated Sequon Adjacent to the Cleavage Site Is of Minor Importance for IL-6R Cleavage

Having excluded that glycosylation is important for the signaling capacity of the IL-6R, we sought to analyze a possible role for the glycans in terms of IL-6R proteolysis. We first concentrated on the N-/O-glycan pair on Asn-350/Thr-352 adjacent to the ADAM10/17 cleavage site and employed an in vitro cleavage assay. Previous attempts to cleave an IL-6R peptide with recombinant ADAM17 gave mixed results [25, 39]. We synthesized ten different glycopeptides containing either the wild-type sequence (QD) or the Asp358Ala mutation (QA) and incubated them with recombinant ADAM17.

**Fig 7:**
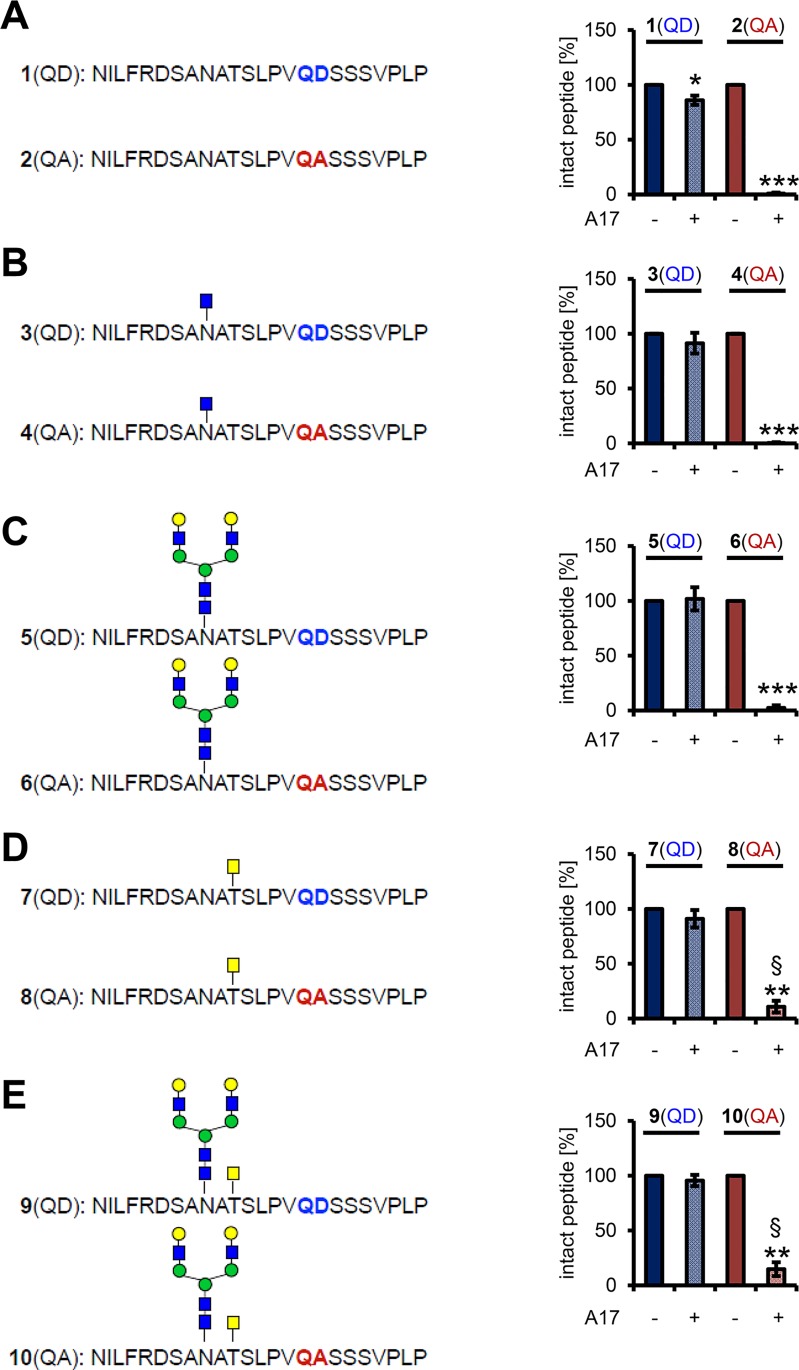
The N- and O-glycosylated sequon adjacent to the cleavage site is of minor importance for IL-6R cleavage. (A) Amino-acid sequence of the two unglycosylated peptides 1(QD) and 2(QA). Peptides were incubated either with or without recombinant ADAM17 for 16 h at 37°C, and the amount of cleaved peptide was analyzed by reversed-phase (RP) chromatography. (B) Amino-acid sequence of the two glycopeptides 3(QD) and 4(QA), which have a GlcNAc attached to the amino-acid residue corresponding to Asn-350 of the IL-6R. Cleavage was determined as described in panel (A). (C) Amino-acid sequence of the two glycopeptides 5(QD) and 6(QA), which have a biantennary N-glycan attached to the amino-acid residue corresponding to Asn-350 of the IL-6R. Cleavage was determined as described in panel (A). (D) Amino-acid sequence of the two glycopeptides 7(QD) and 8(QA), which have a GalNAc attached to the amino-acid residue corresponding toThr-352 of the IL-6R. Cleavage was determined as described in panel (A). (E) Amino-acid sequence of the two glycopeptides 9(QD) and 10(QA), which have a biantennary N-glycan attached to the amino-acid residue corresponding to Asn-350 and a GalNAc attached to the amino-acid residue corresponding toThr-352 of the IL-6R. Cleavage was determined as described in panel (A). All data shown are the mean ± SD from three independent experiments (*p < 0.05, **p < 0.01, ***p < 0.001, compared to the untreated peptide; ^§^*p* < 0.05, compared to the cleaved nonglycosylated peptide).

Whereas the unglycosylated peptide 1(QD) was little but still significantly cleaved (*p* < 0.05, Fig 7A), the corresponding peptide 2(QA) was cleaved completely (1.0% ± 1.0% remaining, *p* < 0.001, Fig 7A). Addition of an N-Acetylglucosamine (GlcNAc) on the residue corresponding to Asn-350 reduced proteolysis of peptide 3(QD) (91.4% ± 9.4% intact) but did not alter processing of peptide 4(QA) (0.4% ± 0.7%, Fig 7B). Interestingly, addition of a larger biantennary N-glycan blocked cleavage of the 5(QD) peptide but did not alter processing of the 6(QA) peptide (Fig 7C). Also of interest, addition of an N-Acetylgalactosamin (GalNAc) to the amino-acid residue corresponding to Thr-352 decreased cleavage of the **7**(QD) peptide (91% ± 8%) but also decreased shedding of the 8(QA) peptide compared to the unglycosylated peptide (10.8% ± 5.4% intact, *p* < 0.05, Fig 7D). Importantly, we have detected GalNAc endogenously on the sIL-6R at Thr-352, but not GlcNAc (Fig 2D and S1A–S1D Fig). When we combined the biantennary N-glycan with the GalNAc, cleavage of 9(QD) was again blocked, but 14.7% ± 6.3% of the 10(QA) peptide was intact, suggesting an additive effect of the two glycans (*p* < 0.05, Fig 7E). Thus, in line with a recent report [25], we can detect an inhibitory influence of the glycosylation on the capacity of ADAM17 to cleave the IL-6R peptide, but overall, the influence appears to be rather small.

### An N-Glycan in Domain D1 Serves as a Protease-Regulatory Exosite

**Fig 8:**
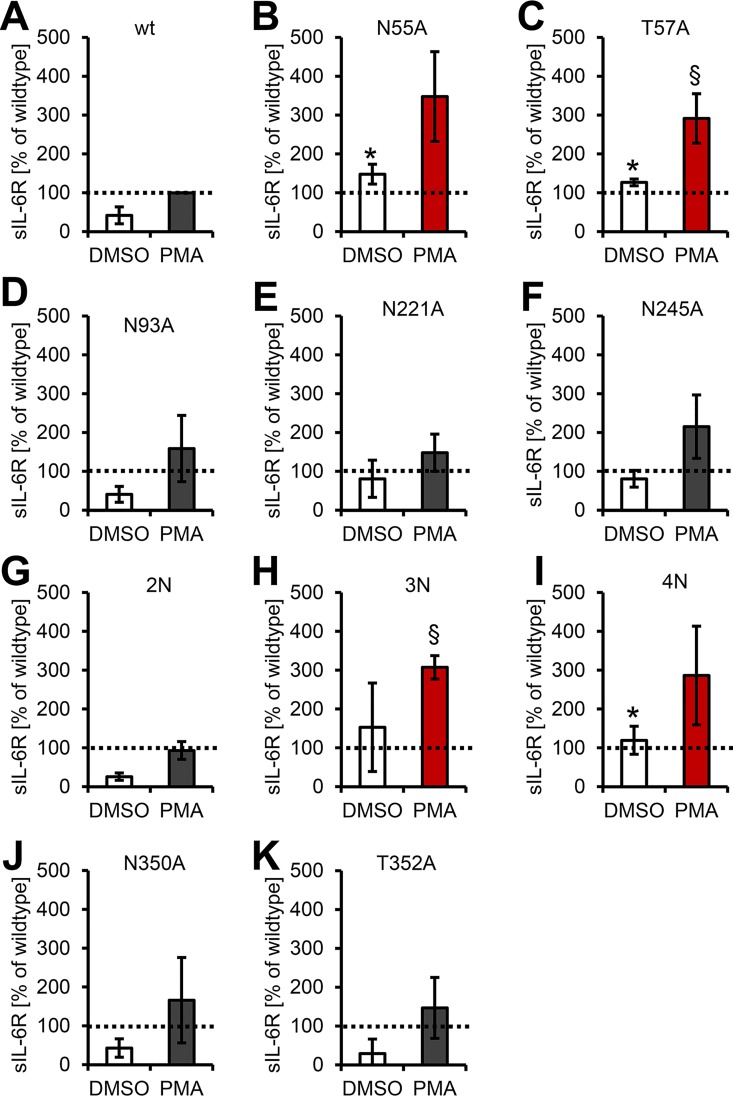
The Asn-55 N-glycan in domain D1 is a protease-regulatory exosite. (A–K) PMA-mediated ectodomain shedding of IL-6R variants lacking single or multiple N-glycans. Experiments were performed with stably transduced Ba/F3-gp130 cell lines. The IL-6R variant is indicated above the respective diagram. Data shown are the mean ± SD from three independent experiments, which were compared to wild-type IL-6R (*p < 0.05, compared to DMSO-treated Ba/F3-gp130-IL-6R cells; ^§^*p* < 0.05, compared to PMA-stimulated Ba/F3-gp130-IL-6R cells).

In order to analyze the role of the other N-glycosylation sites, we used the stably transduced Ba/F3-gp130 cell lines and activated ADAM17-mediated shedding with PMA. We set the amount of sIL-6R shed from wild-type cells to 100% (Fig 8A) and calculated all other values accordingly. Surprisingly, mutation of Asn-55 enhanced PMA-induced shedding to 348% ± 116%, indicating that this N-glycan serves as a protease-regulatory exosite (Fig 8B). Also, unstimulated shedding was significantly increased (148% ± 25%, *p* < 0.05), and the amount was even higher than the stimulated shedding of the wild-type IL-6R, despite equal expression of both receptors (S6A Fig). In order to prove that indeed the glycan and not the asparagine residue is responsible for this effect, we mutated Thr-57, which also leads to the loss of the glycan at Asn-55, and observed a comparable increase in IL-6R shedding (291% ± 64% and 126% ± 9%, both *p* < 0.05, Fig 8C). Mutation of Asn-93 or Asn-221 did not enhance sIL-6R generation (Fig 8D and 8E). We observed slightly enhanced shedding when we mutated Asn-245 (215% ± 82%, Fig 8F). Combined mutation of Asn-93 and Asn-245 (2N) did not enhance proteolysis (Fig 8G), but the 3N and 4N variants, in which Asn-55 was mutated, showed increased IL-6R shedding by ADAM17 (Fig 8H and 8I). In line with the peptide cleavage assays (Fig 7), mutation of the N-/O-glycosylated sequon adjacent to the cleavage site only slightly enhanced proteolysis (Fig 8J and 8K). In conclusion, the N-glycan on Asn-55 within the D1 domain of the IL-6R functions as an exosite, which reduces the induced and constitutive proteolysis of the IL-6R.

### An IL-6R Devoid of All N-Glycans Is Defective in ADAM17-Mediated Proteolysis

**Fig 9:**
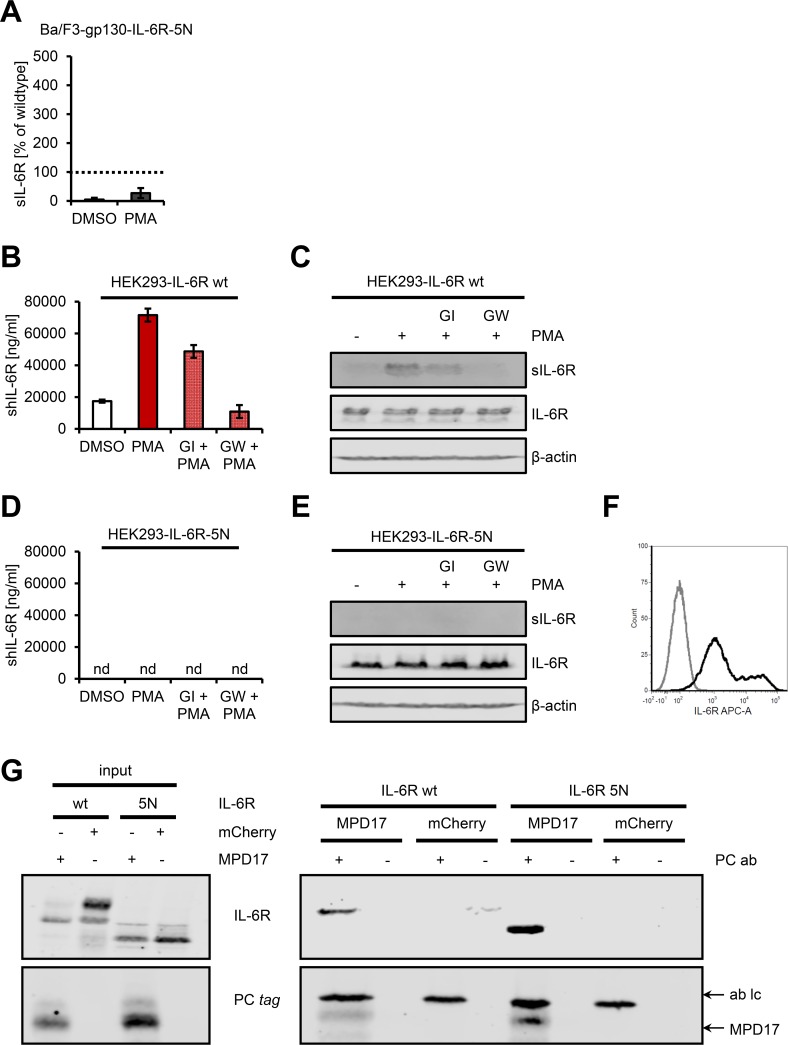
An IL-6R devoid of all N-glycans is defective in ADAM17-mediated proteolysis. (A) PMA-mediated ectodomain shedding of IL-6R-5N with a stably transduced Ba/F3-gp130 cell line. Data shown are the mean ± SD from three independent experiments compared to wild-type IL-6R. (B) HEK293 cells were transiently transfected with a cDNA encoding wild-type IL-6R. Cells were pretreated with the ADAM10-specific inhibitor GI or the combined ADAM10/ADAM17-specific inhibitor GW for 30 min and then stimulated with 100 nM PMA for 2 h as indicated. The amount of sIL-6R in the cell supernatant was determined by ELISA. One out of three experiments with similar outcomes is shown (mean ± SD, *n* = 3). (C) The experiment was performed as described under (B), but sIL-6R was precipitated from cell supernatant and analyzed by western blot. Furthermore, the cells were lysed, and IL-6R expression in the lysates was also determined by western blot. β-actin served as loading control. One experiment out of three with similar outcomes is shown. (D, E) The experiment was performed as described in (C) and (D), but HEK293 cells were transiently transfected with a cDNA encoding IL-6R-5N. nd, not detected. (F) Cell-surface expression of the IL-6R-5N variant on transiently transfected HEK293 cells analyzed via flow cytometry (black histogram). The control staining is shown in gray. (G) HEK293 cells were transiently transfected with expression plasmids encoding wild-type IL-6R or IL-6R-5N in combination with either an expression plasmid encoding mCherry or MPD17-CANDIS. After 48 h, cells were lysed and the MPD17-CANDIS precipitated via its protein C (PC) tag. The expression of all proteins (input) and the interaction of the IL-6R variants with MPD17-CANDIS were analyzed via western blot. One experiment out of two with similar outcomes is shown.

Finally, we analyzed proteolysis of the 5N variant, which lacks all N-glycans. Surprisingly, we detected significantly reduced PMA-induced shedding (27.2% ± 17.2%, Fig 9A), and although the 5N mutant showed less expression at the cell surface compared to the wild type (S6A Fig), this alone cannot explain the reduced proteolysis. To verify this observation, we transiently transfected HEK293 cells side by side with cDNAs encoding wild-type IL-6R or the 5N mutant and analyzed shedding by ELISA and western blot. PMA strongly induced shedding of wild-type IL-6R (Fig 9B and 9C), but we could not detect proteolysis of the 5N mutant in HEK293 cells either by ELISA (Fig 9D) or by western blotting (Fig 9E), although the IL-6R-5N was transported to the cell surface (Fig 9F). We have previously shown that the membrane-proximal domain (MPD17) and especially the conserved ADAM seventeen dynamic interaction sequence (CANDIS) region of ADAM17 are important for substrate recognition [26, 40], and we thus wondered whether the IL-6R-5N variant is still able to bind to ADAM17. As shown in Fig 9G, both IL-6R and IL-6R-5N could be efficiently coimmunoprecipitated with MPD17-CANDIS, indicating that the glycans are not mandatory for the IL-6R/ADAM17 interaction. Thus, although the IL-6R-5N mutant contained the unaltered cleavage site and is able to bind to ADAM17, the protease is nevertheless not able to shed the IL-6R.

## Discussion

Soluble cytokine receptors play pivotal roles in health and disease. As long as the ligand-binding domains are retained within the soluble protein, they have similar affinities towards their ligands as the membrane-tethered counterparts. Most of them have antagonistic properties, because they compete with the membrane-bound receptors for the same cytokine, and cytokines bound to soluble receptors are no longer able to bind to their cognate target cells in order to activate them. The most prominent examples are the soluble receptors for tumor necrosis factor alpha (TNFα) [41]. An Fc-fusion protein of the extracellular part of tumor necrosis factor alpha receptor 2 (TNFR2)/p75 is approved under the name etanercept for the treatment of rheumatoid arthritis and psoriasis [42].

A rare example of an agonistic soluble receptor is the sIL-6R. Surprisingly, despite its importance in terms of disease and therapy, little is known about the mechanisms that lead to sIL-6R *generation in vivo*. In the present study, we confirm the presence of the ds-sIL-6R in human serum and detect for the first time a second form of the sIL-6R with a novel C-terminus generated by proteolytic cleavage. Interestingly, the cleavage event occurs between Pro-355 and Val-356 within the stalk region of the IL-6R. This cleavage site does not match the one previously described by Müllberg et al., who reported cleavage two amino-acid residues further downstream between Gln-357 and Asp-358 [14]. However, cleavage site profiling of ADAM17 as well as data in the MEROPS database confirms a strong preference for a valine at the P1′ site and disfavors an aspartic acid at that position [23, 24], making the reported cleavage by Müllberg et al. between a glutamine and an aspartic acid in retrospect rather unlikely. Moreover, we also consistently found ADAM-mediated cleavage at Pro-355/Val-356 when we treated HEK293 cells overexpressing the IL-6R with PMA or when we incubated a peptide of the IL-6R stalk with the recombinant catalytic domain of ADAM17. We cannot absolutely exclude that exoprotease activity led to trimming of the C-terminal peptide in vivo or during sample preparation, which might confound the identification of the cleavage site via LC-MS. The different technique to determine the cleavage site used in Müllberg et al. [14] might also account for the difference. However, our mutational analysis of the novel cleavage site, which suggests that substitution of Val-356 by glycine or glutamic acid is sufficient to render the IL-6R resistant towards ADAM-mediated proteolysis, further corroborates this finding.

The major genetic determinant of human sIL-6R serum levels is a single nucleotide polymorphism (rs2228145), which leads to the exchange of Asp-358 to an alanine residue [38]. Homozygous carriers have strongly increased sIL-6R serum levels, which reduces their risk of suffering from coronary heart disease [43, 44]. Although differential splicing of the *IL6R* mRNA is increased in these individuals [45], the majority of the sIL-6R is nevertheless generated by an alternative mechanism. We have shown previously that an IL-6R containing the Asp358Ala mutation is more efficiently shed by ADAM10 and ADAM17, which we thought was caused by the exchange of an aspartic acid to an alanine residue at the P1′ position of the cleavage site, which makes the IL-6R a better substrate for both metalloproteases [21, 23]. Because we have now determined the cleavage site between Pro-355 and Val-356, this explanation for the observed effects of the Asp358Ala mutation has to be revised. However, the cleavage site profiling by Tucher et al. clearly shows a preference for an alanine over an aspartic acid residue also at the P3′ position, which explains the observed increase in proteolysis of the Asp358Ala mutant [23].

We have further used the LC-MS approach to determine all N- and O-glycans of the sIL-6R and investigated their functional role(s) in terms of signaling and proteolysis. Surprisingly, mutation of all glycan sites did not significantly alter the signaling capacity of the IL-6R, as shown by the IL-6-dependent proliferation of stably transduced Ba/F3-gp130 cells. This is in contrast to other cytokine receptors like epidermal growth factor receptor (EGFR) and granulocyte macrophage colony-stimulating factor receptor (GM-CSFR) [10, 11], in which N-linked glycosylation is essential for ligand binding. A mutant devoid of all glycans of the IL-6 β-receptor gp130 was still able to signal, but the transport and the stability of the protein were severely compromised [9]. However, the transport of the unglycosylated IL-6R mutant to the cell surface was only marginally affected, and the protein turnover compared to the wild-type IL-6R was not altered at all. Further experiments will show whether this is also true for the other two α-receptors of the IL-6 family, namely the IL-11R and the ciliary neurotrophic factor receptor (CNTFR) [3].

N- and O-linked glycosylation are common post-translational modifications that have been described for a variety of ADAM substrates besides the IL-6R, e.g., for TNFα [46] or transforming growth factor alpha (TGFα) [47]. Substrates with and without glycans have also been used to generate novel ADAM17-specific inhibitors [48]. Recently, it was shown that O-glycosylation near the cleavage site affects ADAM17-mediated proteolysis of small peptides of several ADAM substrates, among them the IL-6R [25]. Here, we show that the IL-6R is indeed O-glycosylated on Thr-352 in vivo and confirm that this glycan reduces cleavage of an IL-6R peptide in conjunction with an N-linked glycan on Asn-350. Cell-based assays, however, revealed a rather small impact of the N-/O-glycosylated sequon adjacent to the cleavage site on ADAM17-mediated proteolysis. In contrast, an N-linked glycan on Asn-55, located in the D1 domain of the IL-6R far away from the cleavage site, was surprisingly identified as a protease-regulatory exosite, whose deletion caused increased shedding of the IL-6R. Importantly, this glycan on Asn-55 has no role in IL-6-mediated signaling. IL-6 binds to the CBM of the IL-6R located in domains D2 and D3, and the D1 domain is not in contact with IL-6 or gp130. Consequently, Hyper-IL-6 does not contain this domain [49], and a membrane-bound IL-6R variant lacking D1 is biologically active [50]. Furthermore, the solved crystal structure of the hexameric IL-6/IL-6R/gp130 complex contains an IL-6R that only consists of the domains D2 and D3 [51]. Thus, our data suggest that the glycan on Asn-55 has a unique role in the regulation of proteolysis but is dispensable for signaling of IL-6.

Furthermore, an IL-6R mutant without any N-glycans reached the cell surface and was able to mediate IL-6-dependent signaling, but its shedding was severely impaired. It is currently unclear why this IL-6R variant, which contains the cleavage site and is able to coprecipitate with the protease via CANDIS, is nevertheless largely resistant towards proteolysis. A possible explanation is cooperativity between the individual glycosylation site, and simultaneous loss of all five N-glycans results in structural changes within the IL-6R ectodomain that disturb cleavage by the protease but do not perturb IL-6-dependent signaling.

In summary, we identify a soluble form of the IL-6R in human serum that is generated by a protease in vivo and map the cleavage site by mass spectrometry. We mutate this cleavage site to generate IL-6R variants that are resistant towards ADAM-mediated cleavage. Furthermore, we map the occupancy of all N- and O-glycosylation sites of the sIL-6R and find that glycosylation is dispensable for trafficking, stabilization, and signaling of the IL-6R but is an important regulatory mechanism in terms of proteolysis.

## Materials and Methods

### Ethics Statement

Ethic approval for this study was obtained from the institutional review board of the Medical Faculty of Kiel University (study #D 515/13).

### Cells and Reagents

Ba/F3-gp130 cells were obtained from Immunex (Seattle, Washington, United States; [52]). The HEK293 cells were from DSMZ GmbH (Braunschweig, Germany), and the Phoenix-Eco cells from U. Klingmüller (DKFZ, Heidelberg, Germany). HEK293-EBNA cells have been described previously [53]. All cells were grown under standard conditions in DMEM high-glucose culture medium (Sigma-Aldrich, Deisenhofen, Germany). The DMEM was supplemented with 10% fetal bovine serum, penicillin (60 mg/l), and streptomycin (100 mg/l), and the cells were kept at 37°C and 5% CO_2_ in a standard incubator with a water-saturated atmosphere. The Ba/F3-gp130 cells were cultured using 10 ng/ml of the recombinant IL-6/sIL-6R fusion protein Hyper-IL-6. After stable transduction (see below) with IL-6R variants, the Ba/F3-gp130 cells were cultured with 10 ng/ml recombinant human IL-6 instead of Hyper-IL-6. The anti-hIL-6R mAb 4–11 was described previously [31]; the anti-hIL-6R mAb (sc-661) and the anti-β-actin mAb (sc-47778) were from Santa Cruz Biotechnology (Santa Cruz, California, US). The anti-GAPDH mAb was obtained from Cell Signaling Technology (Frankfurt/M., Germany). The biotinylated pAb goat anti-hIL-6R antibody Baf227 was from R&D Systems (Wiesbaden-Nordenstadt, Germany). The rabbit pAb ds6R that specifically detected the C-terminus of the ds-sIL-6R was generated by Pineda Antikörper-Service (Berlin, Germany). The peroxidase conjugated secondary antibodies were obtained from Pierce (Thermo Scientific, Perbio, Bonn, Germany), and the APC-conjugated anti-mouse monoclonal secondary antibodies for flow cytometry experiments were obtained from Dianova (Hamburg, Germany). PMA and ionomycin were purchased from Sigma-Aldrich (Deisenhofen, Germany). G-418 and puromycin were from Carl Roth (Karlsruhe, Germany). Purified human CG was purchased from Athens Research (Athens, Georgia, US). PNGase F and the Deglyco-Mix were purchased from New England Biolabs GmbH (Frankfurt am Main, Germany). Protein G- and A-agarose-beads were obtained from Merck/Millipore (Darmstadt, Germany), and NHS-Sepharose from GE Healthcare (München, Germany). Concanavalin A-covered sepharose beads were from Sigma-Aldrich (Taufkirchen, Germany).

### Recombinant Proteins

Hyper-IL-6 was produced as described previously [54, 55]. IL-6 was expressed, purified, and refolded as described previously [56]. The recombinant glycosylated catalytic domain (aa Pro-18 to Val-477) of ADAM17 was purchased from Enzo Life Sciences GmbH (Lörrach, Germany). Recombinant sIL-6R and recombinant ds-sIL-6R were expressed in HEK293-EBNA cells as follows: 2 x 10^6^ cells were seeded in 10 cm cell culture dishes and transfected 24 h later using polyethylenimin (PEI) according to the manufacturer’s instructions. Forty-eight h later, expressing cells were selected with 0.25 mg/ml G-418 and 1 μg/ml puromycin for 3 wk. Afterwards, proteins were produced by seeding 4 x 10^6^ cells in a 175 cm² cell culture flask in 20 ml DMEM (supplemented with 10% fetal bovine serum, penicillin [60 mg/l], streptomycin [100 mg/l], G-418 [250 mg/l], and puromycin [1 mg/l]). After the cells reached confluency, the medium was replaced by 20 ml DMEM (supplemented with G-418 [250 mg/l], puromycin [1 mg/l], and 5% Ultra Low IgG FCS from PAN-Biotech [Aidenbach, Germany]). The medium was harvested two times a week, and the cell culture supernatant filtrated using a vacuum filter unit (pore size 22 μM). After devolatilization, recombinant proteins were isolated with a His-Trap FF Crude column (GE Healthcare, Munich) on an Aekta Purifier (GE Healthcare) and eluted with 50 mM and 100 mM imidazole in PBS. The proteins were further purified via size exclusion chromatography on a Superdex 200 column (GE Healthcare).

### Expression Plasmids

Construction of the different expression plasmids encoding IL-6R variants was performed using standard molecular biology techniques with restriction-enzyme-based cloning. The template expression plasmid encoding the human IL-6R in pcDNA3.1 has been described previously [27]. Splicing by overlapping extension (SOE)-PCR was used to mutate single or multiple N- and O-glycosylation sites. Mutations within the protease cleavage site of the IL-6R were performed similarly. For retroviral transduction of Ba/F3-gp130 cells, constructs were subcloned into the pMOWS plasmid [57]. For the production of recombinant sIL-6R variants in HEK293-EBNA cells, a 6xHis-tag was inserted between the signal peptide and the D1 domain at the N-terminus. The ds-sIL-6R construct had a stop codon after the unique C-terminus GSRRRGSCGL, and the sIL-6R construct had a stop codon after Gln-358 (see Fig 1A). The ADAM17-MPD-CANDIS construct was described previously [40].

### Retroviral Transduction of Ba/F3-gp130 Cells

Retroviral transduction of Ba/F3-gp130 cells via Phoenix-Eco cell supernatant has been described previously [57–59]. Cells stably expressing IL-6R were selected with puromycin (1.5 μg/ml) and subsequently cultivated with 10 ng/ml recombinant IL-6 instead of Hyper-IL-6.

### Cell Viability Assay

Proliferation of the different Ba/F3-gp130-IL-6R cell lines in response to IL-6 and Hyper-IL-6 and of Ba/F3-gp130 cells in response to IL-6 and the two different recombinant sIL-6R variants was determined using the Cell Titer Blue Cell viability assay reagent (Promega, Karlsruhe, Germany), following the manufacturer’s protocol as described previously [59]. Values were measured in triplicates per experiment, and relative light units (RLU) obtained after 60 min were normalized by subtraction of control values obtained after 0 min. The maximum proliferation was set to 100%, and all other values were calculated accordingly.

### Collection of Human Serum

Peripheral blood from healthy volunteers was collected by venipuncture and serum isolated via centrifugation.

### Precipitation of sIL-6R from Cell Culture Supernatant

Nine hundred μl of cell culture supernatant was mixed with 100 μl Concanavalin A beads and incubated overnight at 4°C under constant agitation. The beads were washed three times with 1 ml PBS each and boiled at 95°C for 5 min in 25 μl 3x Laemmli buffer (6% SDS, 30% Glycerol, 5% β-mercaptoethanol, 150 mM Tris-HCl [pH 6.8], and 0.2% bromphenol blue). Samples were analyzed by western blot as described below. For mass spectrometry analysis, sIL-6R was precipitated using 4–11 antibodies covalently linked to NHS-beads (25 μl 4-11-beads per 1 ml supernatant). The samples were run on a 10% SDS-PAGE under reducing conditions and stained with Coomassie, and the region corresponding to sIL-6R was sliced out and analyzed by mass spectrometry (see section below). Successful precipitation was confirmed by western blot.

### Immunoprecipitation of sIL-6R from Human Serum

Twenty μl protein G- and 20 μl protein A-agarose-beads were added per 1 ml serum to remove antibodies and incubated overnight under constant agitation. Afterwards, the beads were removed by centrifugation, and sIL-6R was precipitated with 4-11-beads as described above. SDS-PAGE was performed under nonreducing conditions, the gel was stained with Coomassie, and the region corresponding to sIL-6R was sliced out and analyzed by mass spectrometry (see section below). Successful precipitation was confirmed by western blot.

### In-Gel Digestion

After excision of gel bands and slicing them into small cubes (~1 mm^3^), the gel pieces were washed with water and 50 mM ammonium bicarbonate (ABC) and destained (3–4 incubations with 30% acetonitrile [ACN]/50 mM ABC), followed by dehydration (ACN) and drying (10 min at 45°C in a SpeedVac vacuum centrifuge [Eppendorf, Hamburg, Germany]). Disulfide bond reduction was performed with 10 mM dithiothreitol (DTT) for 1 h at 56°C, followed by alkylation of reduced cysteines with 55 mM iodoacetamide (IAA) for 1 h at 25°C in the dark. Finally, gel bands were washed once more with H_2_O and twice with 50 mM ABC followed by dehydration with 100% ACN. Two strategies were applied (i) to identify the C-terminus of sIL-6R and (ii) to map the N-glycosylation sites. For the identification of the C-terminus of sIL-6R, N-glycans were deglycosylated with 50–100 U PNGase F overnight at 37°C in 50 mM ABC. Afterwards, the gel bands were washed once with 50 mM ABC and three times with H_2_O. After dehydration of the gel bands, proteins were in-gel digested overnight with 50–100 ng of the endoproteases chymotrypsin (Sigma-Aldrich, Steinheim, Germany), trypsin, or Asp-N (both from Promega, Madison, WI) in 25 mM ABC containing 50% H_2_^18^O (from Eurisotop, St-Aubin Cedex, France). For the assignment of N-glycosylation sites, the same protocol was performed, but H_2_^18^O was only used during the deglycosylation step, while the proteolysis with chymotrypsin, trypsin, or Asp-N was performed in 25 mM ABC. Samples were acidified with 10% formic acid (FA) to pH 3–4. Peptide extraction was performed in three cycles with (i) 1% FA, (ii) 60% ACN/1% FA, and (iii) 100% ACN by incubating for 15 min at 25°C, with sonication for 2 min in ice-cold water. Between each step, the liquid supernatants were separated from the gel bands and collected in a second reaction tube. Merged supernatants were dried in a vacuum centrifuge, and the samples were reconstituted in 3% ACN/0.1% trifluoroacetic acid (TFA).

### Liquid Chromatography Tandem Mass Spectrometry (LC-MS/MS)

In-gel digested in vitro samples were analyzed on a Dionex Ultimate 3000 nano-HPLC coupled to a LTQ Orbitrap Velos with ETD (Thermo Scientific, Bremen) using an analytical setup described before [60]. After injection, samples were washed on a trap column (Acclaim Pepmap 100 C18, 10 mm × 300 μm, 3 μm, 100 Å, Dionex) for 5 min with 3% ACN/0.1% TFA at a flow rate of 30 μl/min prior to peptide separation using an Acclaim PepMap 100 C18 analytical column (15 cm × 75 μm, 3 μm, 100 Å, Dionex). A flow rate of 300 nL/min using eluent A (0.05% FA) and eluent B (80% ACN/0.04% FA) was used for gradient separation as follows: linear gradient 5%–50% B in 120 min, 50%–95% B in 5 min, 95% B for 10 min, 95%–5% B in 0.1 min, and equilibration at 5% B for 10 min. Spray voltage applied on a metal-coated PicoTip emitter (30 μm tip size, New Objective, Woburn, Massachusetts, US) was 1.25 kV, with a source temperature of 250°C. Full scan MS spectra were acquired from 5 to 145 min between 300 and 2,000 m/z at a resolution of 60,000 at m/z 400 (automatic gain control [AGC] target of 1e6; maximum ion injection time [IIT] of 500 ms). The five most intense precursors with charge states ≥2+ were used (i) for collision-induced dissociation (CID) with fragment ion detection in the ion trap (parameters: normalized collision energy [NCE] of 35%; isolation width of 2 m/z; resolution, AGC target of 1e4 and maximum IIT of 100 ms) and (ii) for higher-energy collisional dissociation (HCD) with fragment detection in the orbitrap with an NCE of 40% (parameters: isolation width of 3 m/z; resolution, 7,500 at m/z 400; AGC target of 1e5 and maximum IIT of 1,000 ms). The precursor mass tolerance was set to 10 ppm, and dynamic exclusion (30 s) was enabled. For all spectra, a lock mass correction was performed using the polysiloxane contaminant signal at 445.120025 m/z.

The serum samples were analyzed on a Dionex Ultimate 3000 nano-UHPLC coupled to a QExactive Plus (both from Thermo Scientific, Bremen). Injected samples were loaded and washed as described above. Peptide separation was performed using an Acclaim PepMap 100 C18 analytical column (50 cm × 75 μm, 2 μm, 100 Å, Dionex) with a flow rate and eluent composition as described above: linear gradient 5%–50% B in 120 min, 50%–90% B in 5 min, 90% B for 10 min, 90%–5% B in 0.1 min, and equilibrating at 5% B for 10 min. Ionization was performed with 2.4 kV spray voltage applied on a noncoated PicoTip emitter (10 μm tip size, New Objective, Woburn, Massachusetts, US) with the source temperature set to 250°C. MS data were acquired from 5 to 115 min with MS full scans between 300 and 2,000 m/z at a resolution of 70,000 at m/z 200 (AGC value of 3e6 and maximum IIT of 100 ms). The ten most intense precursors with charge states ≥2+ were subjected to fragmentation with HCD with NCE of 28% (isolation width of 3 m/z; resolution, 17,500 at m/z 200; AGC target of 1e5 and maximum IIT of 100 ms). Dynamic exclusion for 45 s was applied with a precursor mass tolerance of 10 ppm. Lock mass correction was performed as described above. Acquired spectra were first searched by computer-assisted database searches using Proteome Discoverer 1.4.1.14 with the search engines SequestHT (Thermo Scientific) and Mascot (Matrix Science, Boston, Massachusetts). Searches were performed against the full human protein database including isoforms (downloaded from Uniprot, 17 July 2012, appended with common contaminants and enzymes used) using the following settings: semienzyme specificity; three missed cleavage sites; mass tolerances of 10 ppm for precursors and for fragment masses 0.02 Da (HCD) and 0.5 Da (CID); static modifications: carbamidomethylation on Cys; and dynamic modifications: oxidation of Met, deamidation of Asp, and ^18^O-incorporation at peptides C-termini. Beside automated database searches, manual MS/MS-spectra interpretation was performed.

### Ectodomain Shedding Assays

Analysis of IL-6R ectodomain shedding in transiently transfected HEK293 cells or stably transduced Ba/F3-gp130 cell lines by ADAM10 and ADAM17 has been described elsewhere [7, 27]. Analysis of IL-6R proteolysis by CG has been described previously [34].

### Synthesis of Glycosylated IL-6R Peptides 1–10

Solid phase peptide synthesis was carried out on a PTI Tribute peptide synthesizer using Trt-ChemMatrix resin (PCAS Biomatrix, Quebec, Canada). The resin (500 mg, loading 0.42 mmol/g) was activated with 10% acetyl bromide in dichloromethane (DCM) over 3 h and washed with absolute dichloromethane. The activated resin was reacted with the first amino-acid (5 eq.) and DIPEA (5 eq.) in DCM. After 16 h, the resin was washed with NMP and DCM and quenched with a solution of DCM/MeOH/DIPEA (17:2:1) (3x2 min). The resin was subsequently washed with DCM and dried. Peptide synthesis was carried out automatically using 5 eq. of Fmoc amino acid, 5 eq. of HCTU and 9 eq. of 0.4 M DIPEA in DMF. The Fmoc group was cleaved with 20% piperidine in DMF.

The building block Fmoc-Thr(Ac3GalNAc)-OH was synthesized according to [61] and incorporated into the peptide manually. Fmoc-Thr(Ac_3_GalNAc)-OH (2 eq.) and HOOBt (2 eq.) were dissolved DCM/DMF (1:1), DIC (2 eq.) was added, and after 20 min at ambient temperature, the mixure was reacted with the resin for 2 h. On resin, deacetylations were carried out with 5% hydrazine hydrate in DMF. On resin, deallylations and subsequent couplings with GlcNAc amine or the biantennary nonasaccharide amine were carried out according to [62]. The peptides and glycopeptides were cleaved from the resin with a mixture of TFA/TES/H_2_O (96:2:2) for 10 min (3x). The filtrates were concentrated, and the peptides and glycoeptides were precipitated by addition of diethyl ether (10-fold volume) followed by centrifugation. The precipitate was purified by HPLC (Supleco Ascentis C-18, 5μ, 10 x 250 mm, gradient of CH_3_CN-H_2_O with 0.1% TFA).

The N,O-glycosylated peptides 9 and 10 were obtained using Fmoc-Asp(PhiPr)OH [63]. The O-glycosylated peptide was cleaved from the resin with 20% HFIP in DCM for 30 min. After removal of the solvent, the residue was reacted with 6 eq. of *t-*butylcarbazate, 4 eq. of Cl-HOBt, and 4 eq. of DIC at 0°C. The mixture was warmed to ambient temperature and was subsequently (after 24 h) purified by flash chromatography (DCM/MeOH) (0%–15% MeOH). For removal of the PhiPr group, the protected hydrazide was dissolved in DCM containing 1% of TFA. After 30 min, the solution was extracted with dilute KHCO_3_, dried, and concentrated. The aspartylation of the O-glycopeptides was carried out with 1.5 eq. of nonasaccharide amine according to [62] in DMF-DMSO (1:1) using 4 eq. of DIPEA, 2.5 eq. of HOAt, and 2.5 eq. of HATU. After 24 h, the reaction mixture was deprotected by addition of the 20-fold volume of TFA/TES/H_2_O (96:2:2). The mixture was concentrated to half the volume after 2 h. Diethyl ether (10-fold volume) was added, and the precipitated glycopeptide was treated with hydrazine hydrate (5% in CH_3_CN-H_2_O 1:9), dried, and purified by HPLC as described above.

The yields were as follows: 1 (15%), 2 (15%), 3 (16%), 4 (17%), 5 (12%), 6 (13%), 7 (25%), 8 (22%), 9 (25%), 10 (19%).

### In Vitro Cleavage Assay

Two 1.5 ml reaction tubes were filled with 50 μl peptide (50 μM) solved in 25 mM Hepes (pH 9.0), and 0.5 μl of the recombinant catalytic domain of ADAM17 was added to one of the reaction tubes, whereas the other sample remained untreated. All samples were incubated for 16 h at 37°C. Afterwards, 1,150 μl 0.1% TFA was added to each sample, and the peptides were separated on a reverse phase chromatography column (Multo High Bio-200-C18) from Chromatographie Service (Langerwehe, Germany) using a linear gradient elution with a binary solvent system and a flow rate of 1 ml/min. Solvent A consisted of 0.1% TFA, and solvent B consisted of 100% ACN. After peak integration, peak areas for cleaved and noncleaved peptides were determined.

### Deglycosylation of (s)IL-6R

The sIL-6R was precipitated with 4–11 beads from 10 ml supernatant of transiently transfected HEK293 cells after PMA stimulation. Furthermore, about 8 x 10^6^ HEK293 cells, transiently transfected with a cDNA encoding the human IL-6R, were lysed (50 mM Tris, pH 7.5, 150 mM NaCl, 1% Triton X-100, and complete protease inhibitor mixture tablets). Full-length IL-6R was precipitated from the lysate with 4–11 beads. The 4–11 beads were washed three times with PBS, and sIL-6R and full-length IL-6R were eluted in 74 μl H_2_O, 10 μl G7 buffer, 1 μl 10% NP 40, and 2.5 μl β-mercaptoethanol (10 min, 95°C). The eluted volume was divided into three 1.5 ml reaction tubes. The first tube remained untreated, the second was treated with 2 μl PNGase F, and the third was treated with 3 μl Deglyco-Mix. All samples were incubated at 37°C overnight. After addition of 15 μl 5x Laemmli buffer, samples were analyzed by western blot.

### SDS-PAGE and Western Blot

Analysis of (s)IL-6R via SDS-PAGE and western blot has been described previously [34]. The immunoprecipitation of the IL-6R and ADAM17-MPD-CANDIS was performed as described previously [40].

### Flow Cytometry

IL-6R cell surface expression on stably transduced Ba/F3-gp130 cell lines and transiently transfected HEK293 cells was analyzed as described previously [7]. Flow cytometry was performed with a BD Biosciences FACS Canto and FCS Express V3 (De Novo Software, Los Angeles, California, US).

### IL-6R Internalization Assay

In order to analyze internalization of the IL-6R, 1 x 10^7^ cells were washed in PBS, seeded onto a 10 cm dish in serum-free DMEM, and starved for 2 h at 37°C. Afterwards, cells were again washed with PBS, and the IL-6R was labeled with the 4–11 antibody diluted in FACS buffer (1% BSA in PBS). After 1 h incubation on ice, cells were washed and resuspended in 10 ml serum-free DMEM, seeded onto a 10 cm dish, and shifted back to 37°C. At the time points indicated (0–180 min), 1 ml of the cell suspension was taken from the dish, transferred into a 1.5 ml reaction tube, and kept on ice to inhibit further internalization of the IL-6R. After obtaining the last sample, the cells were washed in cold FACS buffer, and the remaining IL-6R on the cell surface was stained with an APC-conjugated anti-mouse mAb. The cells were washed again and analyzed by flow cytometry using the BD Biosciences FACS Canto II (Becton-Dickinson, Heidelberg, Germany).

### Enzyme-Linked ImmunoSorbent Assays

The sandwich ELISA, which detects sIL-6R as well as ds-sIL-6R (4-11/Baf227 antibodies) and can be used to quantify total sIL-6R serum levels, was described previously [21, 31]. To specifically detect ds-sIL-6R (either recombinant or in human serum), a similar approach was used (ds6R/Baf227). Both sandwich ELISAs were performed with streptavidin-horseradish peroxidase (R&D Systems, Minneapolis, Minnesota, US) and the peroxidase substrate BM blue POD (Roche, Mannheim, Germany). The enzymatic reaction was stopped by addition of 1.8 M sulfuric acid, and the absorbance read at 450 nm on a Tecan rainbow reader (Tecan, Crailsheim, Germany).

### Statistical Analysis

Statistical analyses were performed with GraphPad Prism (GraphPad Software, La Jolla, California, US). Data were analyzed by a Student’s *t* test, and the differences were indicated (*^/§^*p* < 0.05; **p < 0.01; ***p < 0.001). The one-sample *t* test was used to compare values to normalized data (e.g., comparison to 100.0 values). *p*-Values were corrected via the Bonferroni method for multiple comparisons.

The numerical data used in all figures are included in S1 Data.

## Supporting Information

S1 Fig Identification of different O-glycan structures on the novel protease-derived sIL-6R from human serum via MS. **(A-E)** MS/MS spectra (HCD) of the C-terminal peptides of the protease-derived sIL-6R identified by manual spectra interpretation. The N-glycan site Asn-350, which is modified to an Asp-350 due to PNGaseF treatment, is shown in green. The different identified O-glycan structures on Thr-352 are shown.
(TIF)

S2 Fig Identification of different O-glycan structures on the ADAM17-derived sIL-6R via MS. **(A-E)** MS/MS spectra (HCD) of the C-terminal peptides of the ADAM17-derived sIL-6R identified by manual spectra interpretation. The N-glycan site Asn-350, which is modified to an Asp-350 due to PNGaseF treatment, is shown in green. The different identified O-glycan structures on Thr-352 are shown.
(TIF)

S3 Fig Identification of different O-glycan structures on the ADAM10-derived sIL-6R via MS. **(A-D)** MS/MS spectra (HCD) of the C-terminal peptides of the ADAM10-derived sIL-6R identified by manual spectra interpretation. The N-glycan site Asn-350, which is modified to an Asp-350 due to PNGaseF treatment, is shown in green. The different identified O-glycan structures on Thr-352 are shown. **(E)** Transiently transfected HEK293 cells were treated either with 100 nM PMA, with 1 μg CG, or left untreated for 2 h. The precipitated sIL-6R from the cell supernatant as well as IL-6R within the cell lysates were analyzed by Western blot. GAPDH served as loading control. One experiment of two performed with similar outcome is shown.
(TIF)

S4 Fig Mutation of Val-356 is sufficient to block ADAM10-mediated proteolysis of the IL-6R and the Asp358Ala variant. **(A)** HEK293 cells were transiently transfected with Expression plasmids encoding the wildtype IL-6R (PV) or the double mutants (IE, DG) as indicated. Cells were either treated with 1 μM ionomycin for 1 h or DMSO as vehicle control. sIL-6R was precipitated from the supernatant with concanavalin A-covered Sepharose beads, and cells were lysed. Both were analyzed via Western blot, and β-actin served as loading control. One out of three experiments with similar outcome is shown. **(B, C)** The experiment was performed as described under (A), but sIL-6R generation was analyzed via ELISA. In (B), the amount of sIL-6R generated after ionomycin-stimulation of the wildtype IL-6R (PV) was set to 100%, and all other values were calculated accordingly. In (C), the amount of sIL-6R without stimulation was considered as constitutive shedding and set to 1 and the increase of sIL-6R was calculated. Data shown are the mean ± SD from at least three independent experiments (*p&lt;0.05, ns = no significant difference). **(D-F)** HEK293 cells were transiently transfected with expression plasmids encoding the wildtype IL-6R (PV) or the single mutants (DV, IV, PE, PG) as indicated. The experiments were performed as described in (A) to (C). **(G) **ADAM10-mediated proteolysis of the IL-6R variants depicted in Fig 4I was analyzed as described in (B). **(H) **Equal numbers of Ba/F3-gp130-IL-6R_DG cells were incubated for 48 h with increasing amounts (0–100 ng/ml) of either IL-6 or Hyper-IL-6. One representative experiment out of three performed is shown (mean ± SD, biological triplicates).
(TIF)

S5 Fig Verification of N-glycosylation sites on the sIL-6R isolated from human serum. **(A-C)** MS/MS spectra (HCD) of the peptides of the sIL-6R isolated from human serum identified via database searching that contain an N-glycosylation site. The site Asn-55, Asn-93 and Asn-221, which are all modified to aspartic acid residues due to PNGase F treatment in the presence of H_2_^18^O-containing buffer, are shown in green.
(TIF)

S6 Fig Cell-surface expression and signaling capacity of different N-glycan mutants. **(A)** Cell-surface expression of the IL-6R on the individual Ba/F3-gp130-IL-6R cell lines. The stably transduced IL-6R mutant is indicated above the histogram. Staining is shown in color, whereas the control staining is shown in gray. One out of three experiments with similar outcome is shown. **(B-D)** Equal numbers of cells of the indicated Ba/F3-gp130-hIL-6R cell line were incubated for 48 h with increasing amounts (0–100 ng/ml) of either IL-6 or Hyper-IL-6. One representative experiment out of three performed is shown (mean ± SD, biological triplicates).
(TIF)

S1 Data Numerical data. Excel spreadsheet containing, in separate sheets, the underlying numerical data for Figure panels 1B, 1C, 1D, 1E, 1F, 1G, 4D, 4E, 4G, 4H, 4J, 4K, 4L, 4M, 6A, 6B, 6C, 6D, 6E, 6F, 6G, 6H, 7A, 7B, 7C, 7D, 7E, 8A, 8B, 8C, 8D, 8E, 8F, 8G, 8H, 8I, 8J, 8K, 9A, 9B, 9D, S4B, S4C, S4E, S4F, S4G, S4H, S6B, S6C and S6D.
(XLSX)

## Abbreviations

ADAM: a disintegrin and metalloproteinase
CANDIS: conserved ADAM seventeen dynamic interaction sequence
CBM: cytokine-binding module
CG: cathepsin G
CNTFR: ciliary neurotrophic factor receptor
CXCL12: C-X-C motif chemokine 12
ds-sIL-6R: differentially spliced sIL-6R
EGF: epidermal growth factor
EGFR: epidermal growth factor receptor
ETD: electron transfer dissociation
GalNAc: N-Acetylgalactosamin
GlcNAc: N-Acetylglucosamine
GM-CSF: granulocyte-macrophage colony-stimulating factor
GM-CSFR: granulocyte macrophage colony-stimulating factor receptor
gp130: glycoprotein 130
HCD: higher-energy collisional dissociation
Ig: immunoglobulin
IL: interleukin
LC-MS: liquid chromatography–mass spectrometry
MPD: membrane-proximal domain
PNGase F: peptide:N-glycosidase F
PMA: phorbol-12-myristate-13-acetate
RP: reversed phase
SD: standard deviation
SDS: sodium dodecyl sulfate
SEM: standard error of the mean
sIL-6R: soluble IL-6 receptor
SNP: single nucleotide polymorphism
TGFαa: transforming growth factor alpha
TNFαa: tumor necrosis factor alpha
TNFR2: tumor necrosis factor alpha receptor 2
