# Supplementary figures and images for: Proteolytic Origin of the Soluble Human IL-6R In Vivo and a Decisive Role of N-Glycosylation
Source: PLoS Biol. 2017 Jan 6;15(1):e2000080. doi: 10.1371/journal.pbio.2000080 (PMC5218472; doi:10.1371/journal.pbio.2000080)

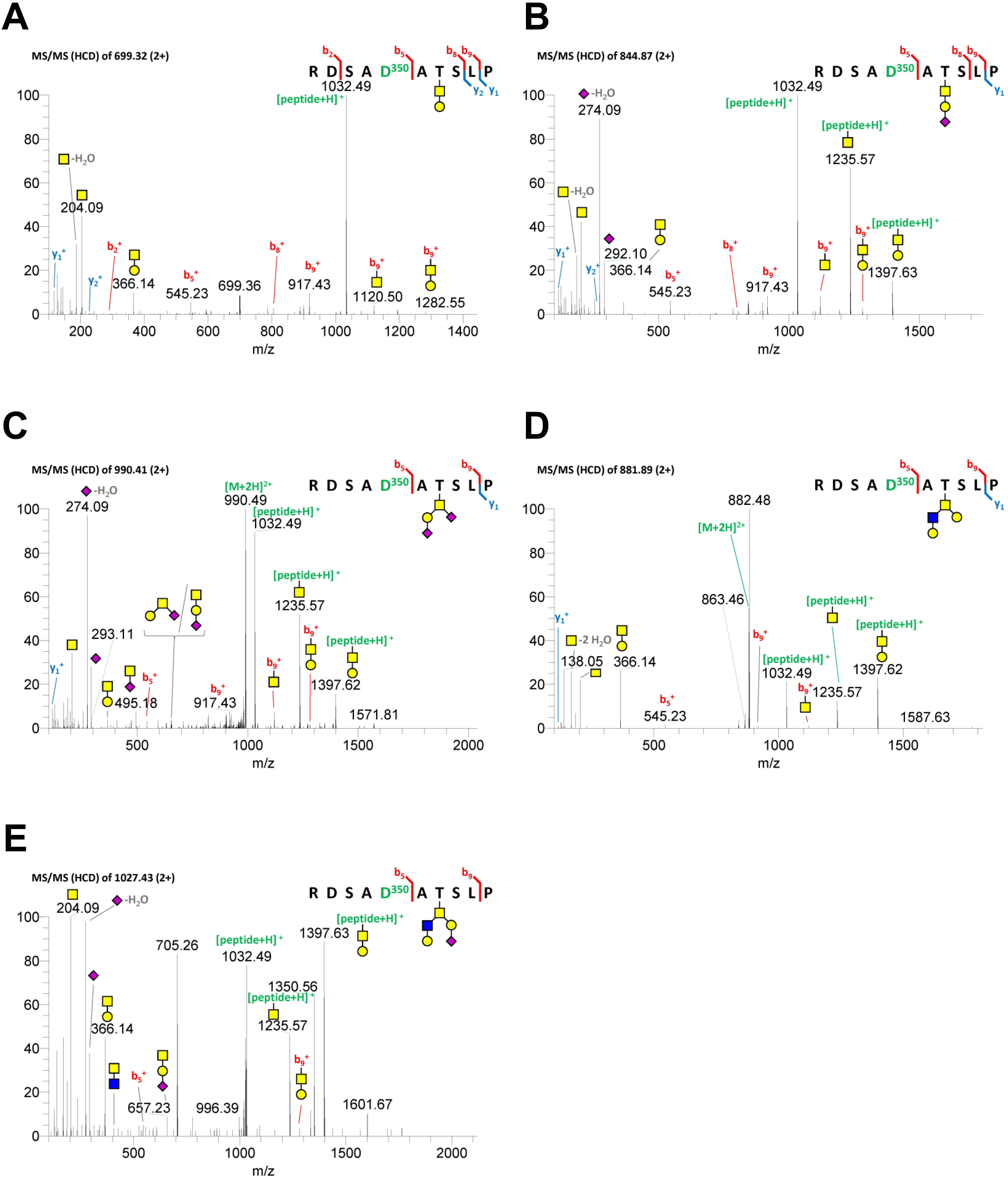

Supplement: S1 Fig — (A-E) MS/MS spectra (HCD) of the C-terminal peptides of the protease-derived sIL-6R identified by manual spectra interpretation. The N-glycan site Asn-350, which is modified to an Asp-350 due to PNGaseF treatment, is shown in green. The different identified O-glycan structures on Thr-352 are shown. (TIF) [file pbio.2000080.s001.tif]

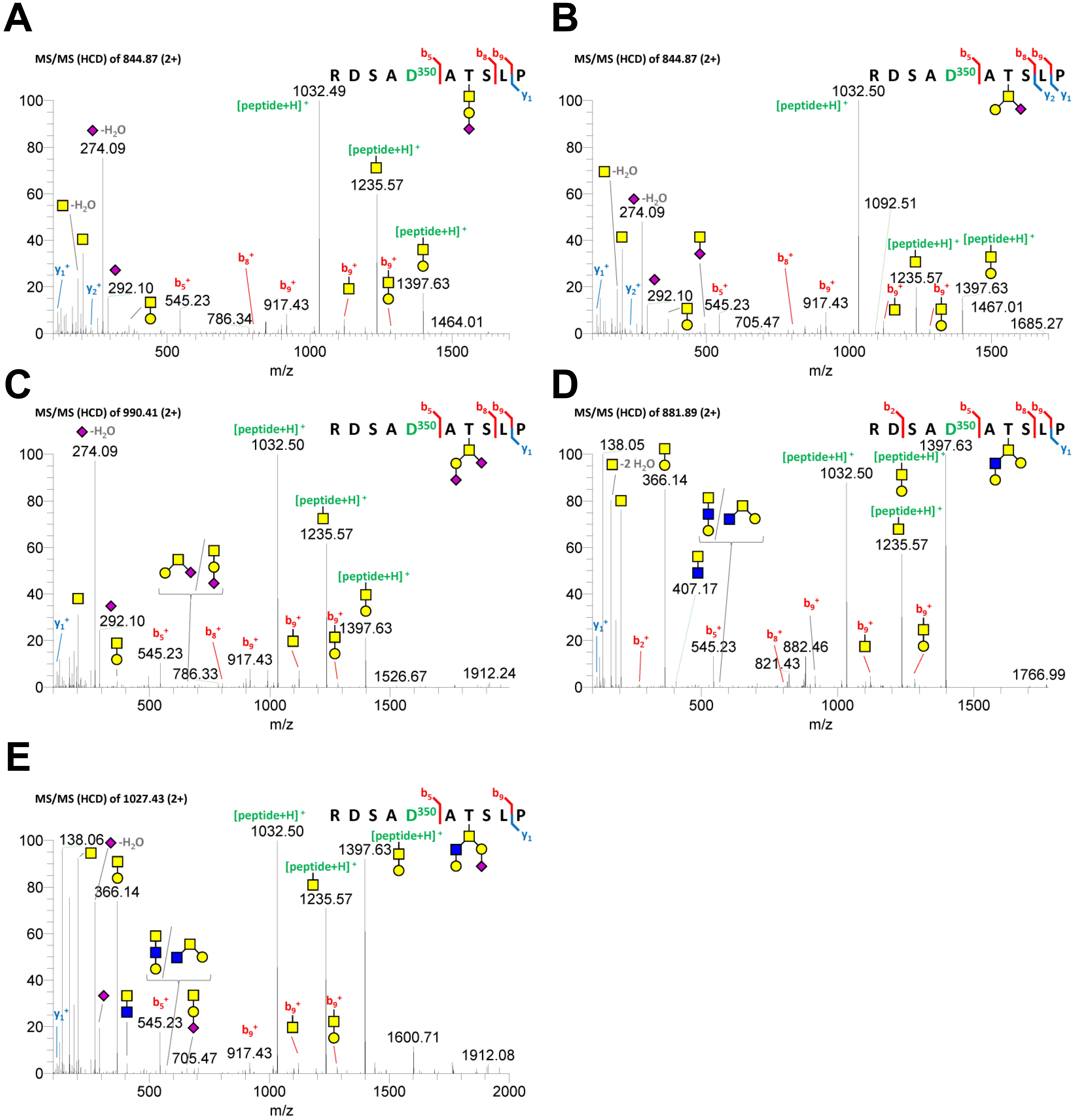

Supplement: S2 Fig — (A-E) MS/MS spectra (HCD) of the C-terminal peptides of the ADAM17-derived sIL-6R identified by manual spectra interpretation. The N-glycan site Asn-350, which is modified to an Asp-350 due to PNGaseF treatment, is shown in green. The different identified O-glycan structures on Thr-352 are shown. (TIF) [file pbio.2000080.s002.tif]

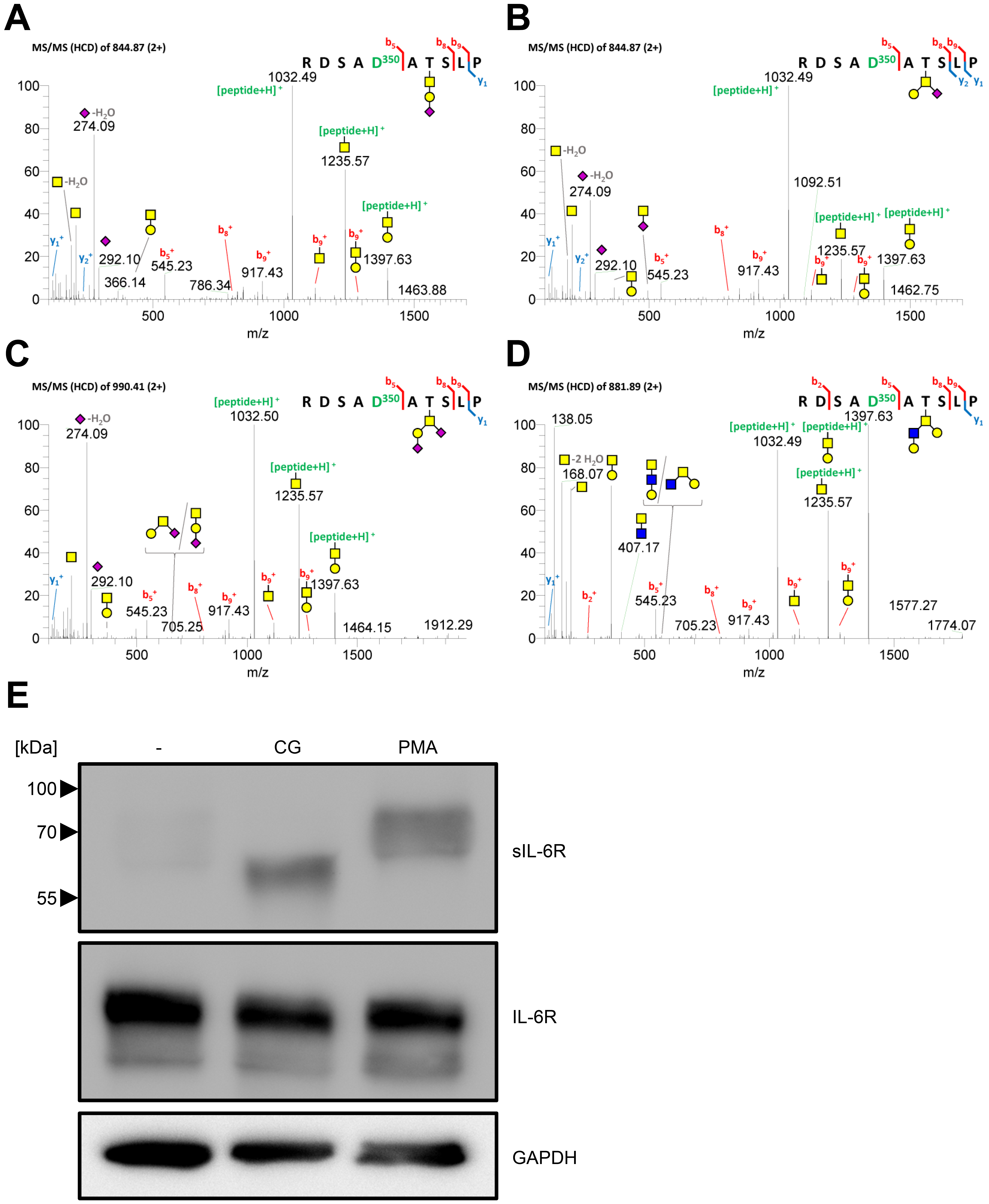

Supplement: S3 Fig — (A-D) MS/MS spectra (HCD) of the C-terminal peptides of the ADAM10-derived sIL-6R identified by manual spectra interpretation. The N-glycan site Asn-350, which is modified to an Asp-350 due to PNGaseF treatment, is shown in green. The different identified O-glycan structures on Thr-352 are shown. (E) Transiently transfected HEK293 cells were treated either with 100 nM PMA, with 1 μg CG, or left untreated for 2 h. The precipitated sIL-6R from the cell supernatant as well as IL-6R within the cell lysates were analyzed by Western blot. GAPDH served as loading control. One experiment of two performed with similar outcome is shown. (TIF) [file pbio.2000080.s003.tif]

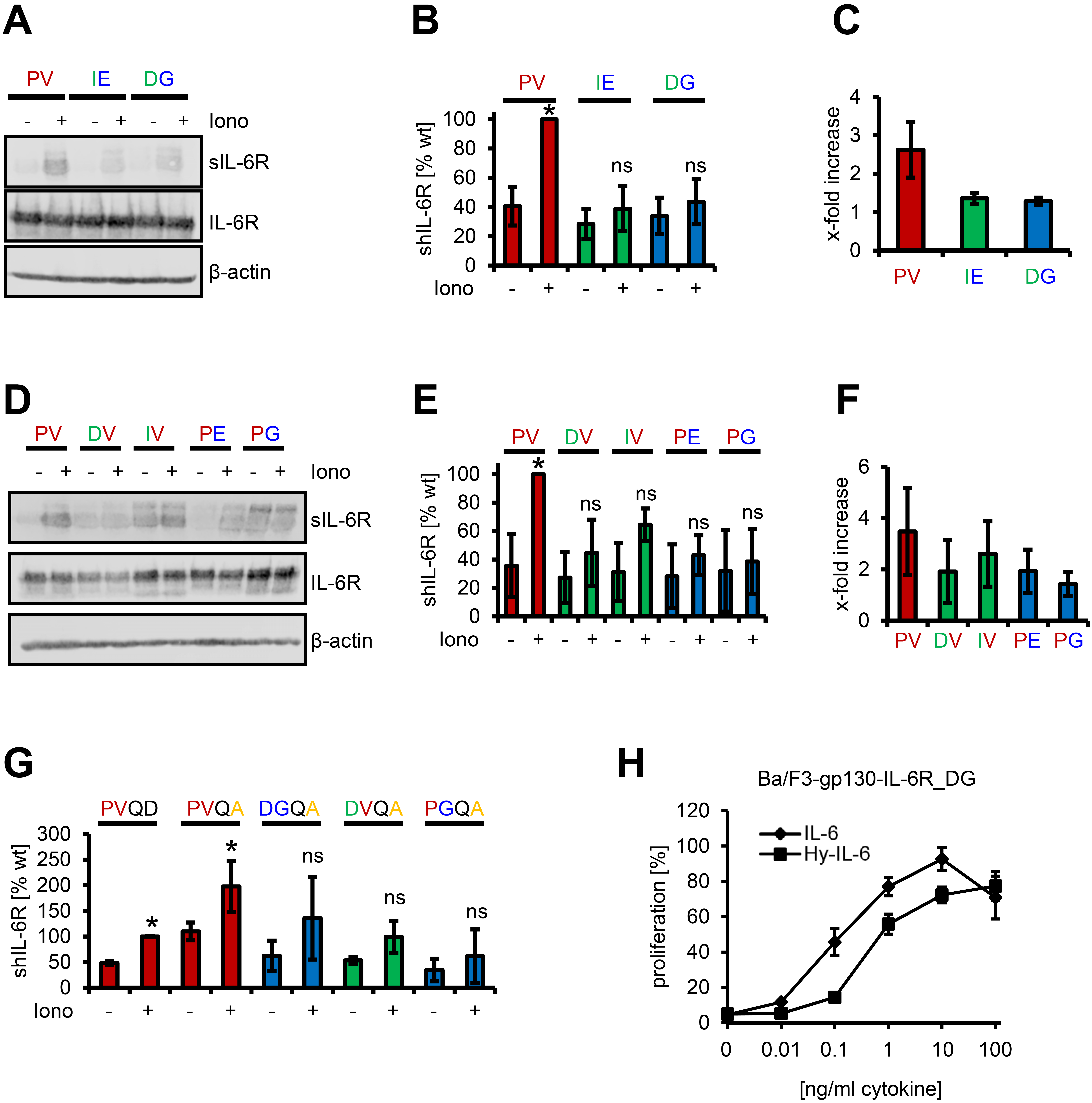

Supplement: S4 Fig — (A) HEK293 cells were transiently transfected with Expression plasmids encoding the wildtype IL-6R (PV) or the double mutants (IE, DG) as indicated. Cells were either treated with 1 μM ionomycin for 1 h or DMSO as vehicle control. sIL-6R was precipitated from the supernatant with concanavalin A-covered Sepharose beads, and cells were lysed. Both were analyzed via Western blot, and β-actin served as loading control. One out of three experiments with similar outcome is shown. (B, C) The experiment was performed as described under (A), but sIL-6R generation was analyzed via ELISA. In (B), the amount of sIL-6R generated after ionomycin-stimulation of the wildtype IL-6R (PV) was set to 100%, and all other values were calculated accordingly. In (C), the amount of sIL-6R without stimulation was considered as constitutive shedding and set to 1 and the increase of sIL-6R was calculated. Data shown are the mean ± SD from at least three independent experiments (*p<0.05, ns = no significant difference). (D-F) HEK293 cells were transiently transfected with expression plasmids encoding the wildtype IL-6R (PV) or the single mutants (DV, IV, PE, PG) as indicated. The experiments were performed as described in (A) to (C). (G) ADAM10-mediated proteolysis of the IL-6R variants depicted in Fig 4I was analyzed as described in (B). (H) Equal numbers of Ba/F3-gp130-IL-6R_DG cells were incubated for 48 h with increasing amounts (0–100 ng/ml) of either IL-6 or Hyper-IL-6. One representative experiment out of three performed is shown (mean ± SD, biological triplicates). (TIF) [file pbio.2000080.s004.tif]

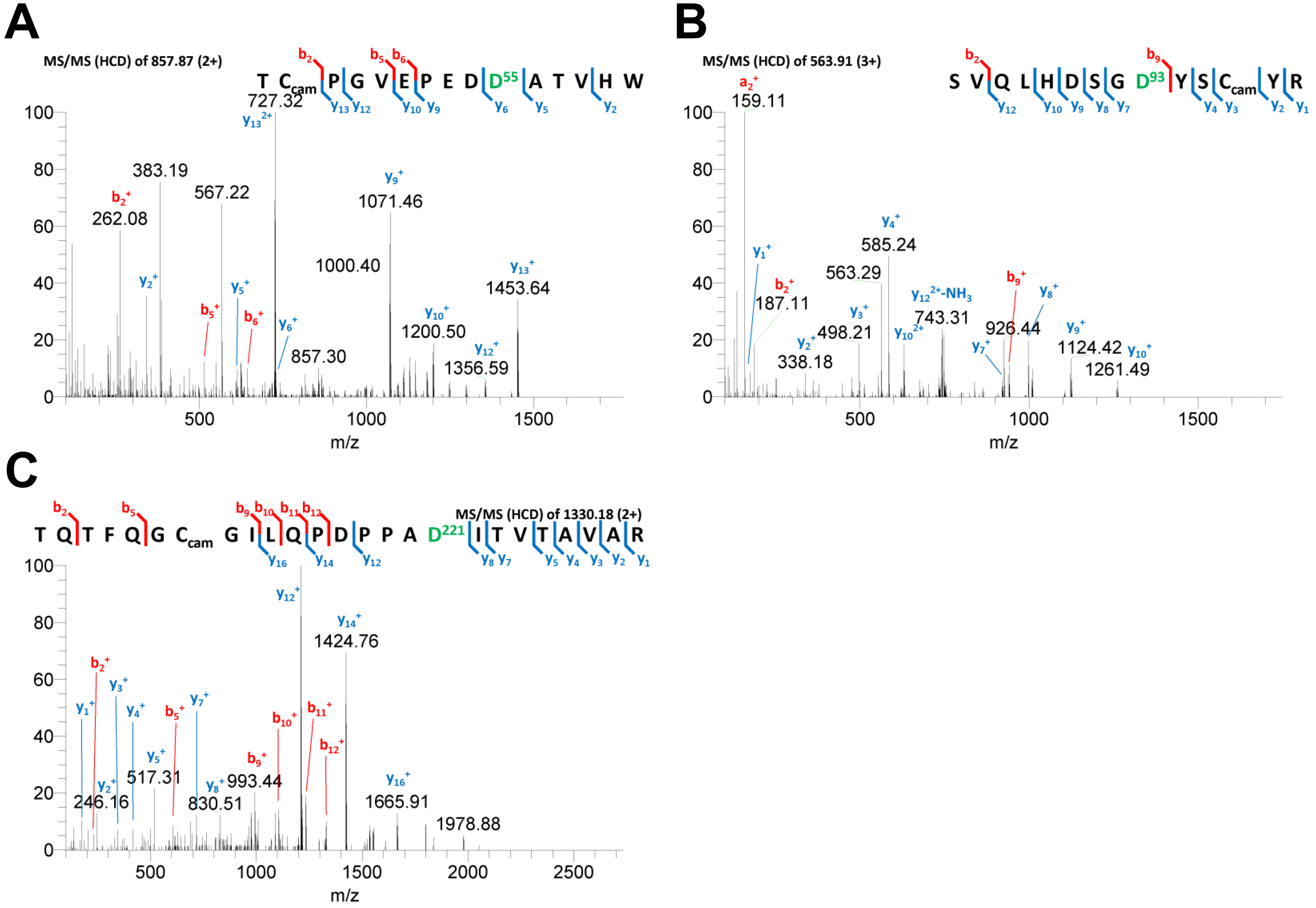

Supplement: S5 Fig — (A-C) MS/MS spectra (HCD) of the peptides of the sIL-6R isolated from human serum identified via database searching that contain an N-glycosylation site. The site Asn-55, Asn-93 and Asn-221, which are all modified to aspartic acid residues due to PNGase F treatment in the presence of H218O-containing buffer, are shown in green. (TIF) [file pbio.2000080.s005.tif]

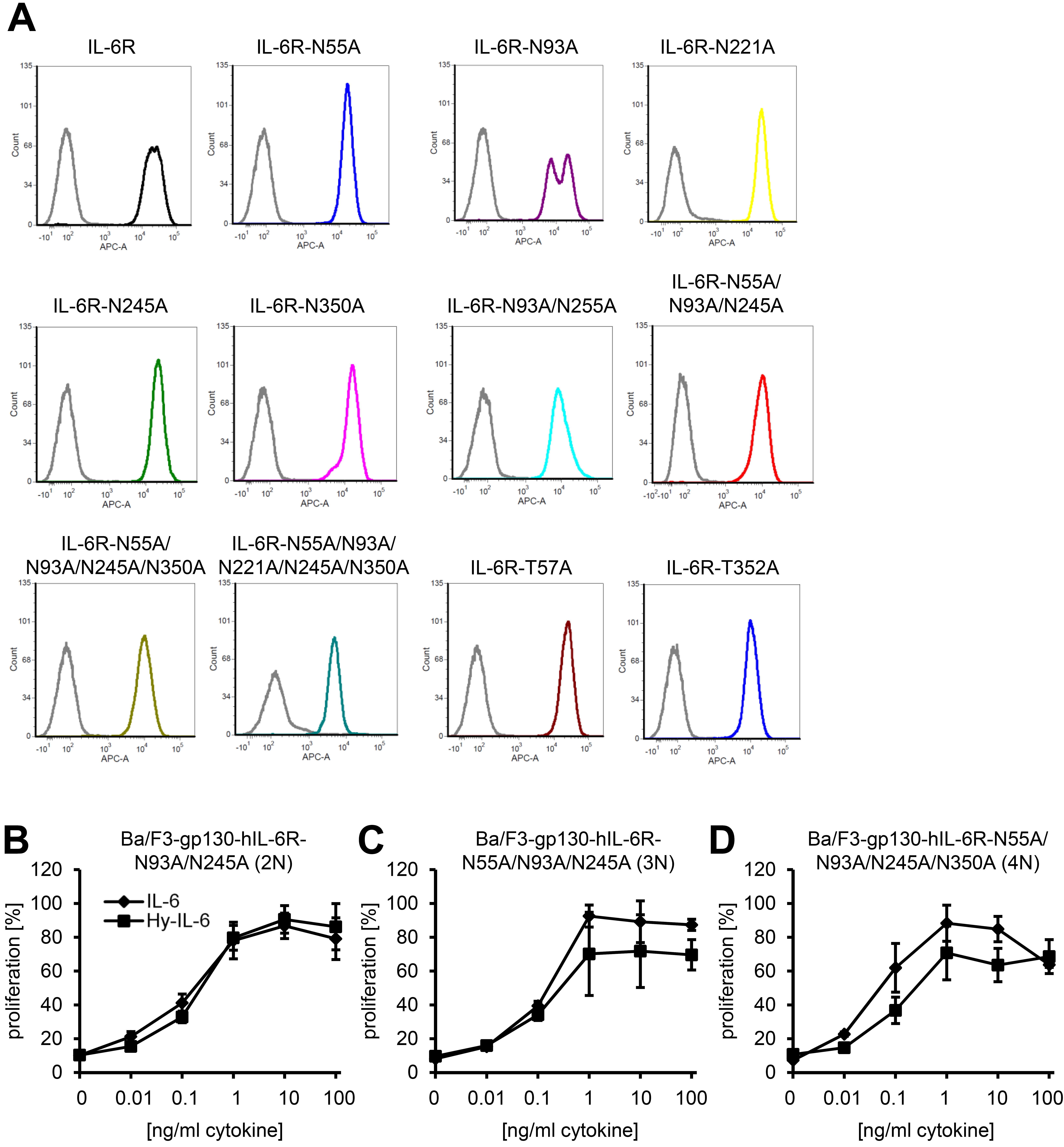

Supplement: S6 Fig — (A) Cell-surface expression of the IL-6R on the individual Ba/F3-gp130-IL-6R cell lines. The stably transduced IL-6R mutant is indicated above the histogram. Staining is shown in color, whereas the control staining is shown in gray. One out of three experiments with similar outcome is shown. (B-D) Equal numbers of cells of the indicated Ba/F3-gp130-hIL-6R cell line were incubated for 48 h with increasing amounts (0–100 ng/ml) of either IL-6 or Hyper-IL-6. One representative experiment out of three performed is shown (mean ± SD, biological triplicates). (TIF) [file pbio.2000080.s006.tif]
